# Risk of bias in studies on prediction models developed using supervised machine learning techniques: systematic review

**DOI:** 10.1136/bmj.n2281

**Published:** 2021-10-20

**Authors:** Constanza L Andaur Navarro, Johanna A A Damen, Toshihiko Takada, Steven W J Nijman, Paula Dhiman, Jie Ma, Gary S Collins, Ram Bajpai, Richard D Riley, Karel G M Moons, Lotty Hooft

**Affiliations:** 1 Julius Centre for Health Sciences and Primary Care, University Medical Center Utrecht, Utrecht University, Utrecht, Netherlands; 2 Cochrane Netherlands, University Medical Center Utrecht, Utrecht University, Utrecht, Netherlands; 3 Centre for Statistics in Medicine, Nuffield Department of Orthopaedics, Rheumatology and Musculoskeletal Sciences, University of Oxford, Oxford, UK; 4 NIHR Oxford Biomedical Research Centre, Oxford University Hospitals NHS Foundation Trust, Oxford, UK; 5 Centre for Prognosis Research, School of Medicine, Keele University, Keele, UK

## Abstract

**Objective:**

To assess the methodological quality of studies on prediction models developed using machine learning techniques across all medical specialties.

**Design:**

Systematic review.

**Data sources:**

PubMed from 1 January 2018 to 31 December 2019.

**Eligibility criteria:**

Articles reporting on the development, with or without external validation, of a multivariable prediction model (diagnostic or prognostic) developed using supervised machine learning for individualised predictions. No restrictions applied for study design, data source, or predicted patient related health outcomes.

**Review methods:**

Methodological quality of the studies was determined and risk of bias evaluated using the prediction risk of bias assessment tool (PROBAST). This tool contains 21 signalling questions tailored to identify potential biases in four domains. Risk of bias was measured for each domain (participants, predictors, outcome, and analysis) and each study (overall).

**Results:**

152 studies were included: 58 (38%) included a diagnostic prediction model and 94 (62%) a prognostic prediction model. PROBAST was applied to 152 developed models and 19 external validations. Of these 171 analyses, 148 (87%, 95% confidence interval 81% to 91%) were rated at high risk of bias. The analysis domain was most frequently rated at high risk of bias. Of the 152 models, 85 (56%, 48% to 64%) were developed with an inadequate number of events per candidate predictor, 62 handled missing data inadequately (41%, 33% to 49%), and 59 assessed overfitting improperly (39%, 31% to 47%). Most models used appropriate data sources to develop (73%, 66% to 79%) and externally validate the machine learning based prediction models (74%, 51% to 88%). Information about blinding of outcome and blinding of predictors was, however, absent in 60 (40%, 32% to 47%) and 79 (52%, 44% to 60%) of the developed models, respectively.

**Conclusion:**

Most studies on machine learning based prediction models show poor methodological quality and are at high risk of bias. Factors contributing to risk of bias include small study size, poor handling of missing data, and failure to deal with overfitting. Efforts to improve the design, conduct, reporting, and validation of such studies are necessary to boost the application of machine learning based prediction models in clinical practice.

**Systematic review registration:**

PROSPERO CRD42019161764.

## Introduction

A multivariable prediction model is defined as any combination of two or more predictors (variables, features) for estimating the probability or risk of an individual having (diagnosis) or developing (prognosis) a particular outcome.^1^ ^2^ ^3^ ^4^ Properly conducted and well reported prediction model studies are essential for the correct implementation of models in clinical practice. Despite an abundance of studies on prediction models, only a limited number of these models are used in clinical practice. As such, many published studies contribute to research waste.^5^ We anticipate that the rise of modern data driven modelling techniques will boost the existing popularity of prediction model studies in the biomedical literature.^6^ ^7^

Machine learning, a subset of artificial intelligence, has gained considerable popularity in recent years. Broadly, machine learning refers to computationally intensive methods that use data driven approaches to develop models that require fewer modelling decisions by the modeller compared with traditional modelling techniques.^8^ ^9^ ^10^ ^11^ Machine learning comprises the two approaches of supervised and unsupervised learning. In the former an algorithm learns to make predictions using previously labelled outcomes, whereas in the latter the algorithm learns to find unexpected patterns using unlabelled outcomes.^12^ Traditional prediction models in healthcare usually resemble the supervised learning approach: datasets used for model development are labelled and the objective is to predict an outcome in new data. Examples of supervised learning include random forest, naïve bayes, gradient boosting machines, support vector machines, and neural networks. Studies on supervised machine learning based prediction models have shown promising and even superior predictive performance compared with conventional statistical techniques; however, recent systematic reviews have shown otherwise.^13^ ^14^ ^15^ ^16^ Although several publications have raised concern about the methodological quality of prediction models developed with conventional statistical techniques,^6^ ^17^ ^18^ a formal methodological and risk of bias assessment of supervised machine learning based prediction model studies across all medical disciplines have not yet been carried out.

Shortcomings in study design, methods, conduct, and analysis might set the study at high risk of bias, which could lead to deviated estimates of the models’ predictive performance.^19^ ^20^ The prediction model risk of bias assessment tool (PROBAST) was developed to facilitate risk of bias assessment and thus provides a methodological quality assessment of primary studies that report on development, validation, or update of prediction models, regardless of the clinical domain, predictors, outcomes, or modelling technique used.^19^ ^20^ Using a prediction model considered at high risk of bias might lead to unnecessary or insufficient interventions and thus affect patients’ health and health systems. Rigorous risk of bias evaluation of prediction model studies is therefore essential to ensure reliable, fast, and valuable application of prediction models.

We conducted a systematic review to assess the methodological quality and risk of bias of studies on supervised machine learning based prediction models across all medical specialties in a contemporary sample of the literature.

## Methods

Our systematic review was reported following the preferred reporting items for systematic reviews and meta-analyses statement.^21^ The review protocol was registered and has been published.^22^

### Identification of prediction model studies

On 19 December 2019, we searched for eligible studies published in PubMed from 1 January 2018 to 31 December 2019 (see supplementary file 1 for search strategy). We restricted our search to obtain a contemporary sample of articles that would reflect current practices in prediction modelling using machine learning. 

Eligible publications needed to describe the development or validation of at least one multivariable prediction model using any supervised machine learning technique that aimed for individualised prediction of risk of patient related health outcomes. Our protocol lists the inclusion and exclusion criteria.^22^ A study was also considered eligible if it aimed to develop a prediction model based on model extension or incremental value of new predictors. No restrictions were applied based on study design, data source, or types of patient related health outcomes. We defined a study to be an instance of machine learning when a non-regression statistical technique was used to develop or validate a prediction model. Therefore we excluded studies using only linear regression, logistic regression, lasso regression, ridge regression, or elastic net. Publications that reported the association of a single predictor, test, or biomarker, or its causality, with an outcome were also excluded, as were publications that aimed to use machine learning to enhance the reading of images or signals or those where machine learning models only used genetic traits or molecular markers as predictors. Other exclusions were systematic reviews, methodological articles, conference abstracts, and publications for which full text was unavailable through our institution. We restricted our search to studies in human participants and articles written in English.

### Screening process

Two independent reviewers, from a group of seven (CLAN, TT, SWJN, PD, JM, RB, and JAAD), screened titles and abstracts. A third reviewer helped to resolve disagreements (JAAD). After potentially eligible studies had been selected, two independent researchers reviewed the retrieved full text articles for eligibility; one researcher (CLAN) screened all articles, and six researchers (TT, SWJN, PD, JM, RB, and JAAD) collectively screened the same articles for agreement. A third reviewer (JAAD) read articles to resolve disagreements.

### Data extraction

We developed a data extraction form based on the four domains: participants, predictors, outcome, and analysis (box) as well as 20 signalling questions as described in PROBAST.^19^ ^20^

Box 1 **Description of domains used in data extraction form**

**Participants domain**

Covers potential biases related to the selection of participants and data sources used

**Predictors domain**

Evaluates potential sources of bias from the definition and measurement of the candidate predictors

**Outcome domain**

Assesses how and when the outcome was defined and determined

**Analysis domain**

Examines the statistical methods that authors have used to develop and validate the model, including study size, handling of continuous predictors and missing data, selection of predictors, and model performance measures

Our extraction form contained three sections for each domain: two to nine specific signalling questions, judgment of risk of bias, and rationale for the judgment. Signalling questions were formulated to be answered as yes or probably yes, no or probably no, and no information. The signalling questions were phrased so that yes or probably yes indicated an absence of bias. Likewise, judgment of risk of bias was defined as high, low, or unclear risk of bias. Also, reviewers provided a rationale for judgment as free text comments.

If a study included external validation, we applied the extraction form to both the development and the external validation of the model. Signalling question 4.5, “Was selection of predictors based on univariable analysis avoided?; 4.8 “Were model overfitting and optimism in model performance accounted for?”; and 4.9 “Do predictors and their assigned weights in the final model correspond to the results from the reported multivariable analysis?” did not apply to external validation. If a study reported more than one model, we applied PROBAST to the recommended model defined by the authors in the article. If the authors did not recommend a model, we selected the one with highest accuracy (in terms of discrimination) as the recommended model. The PROBAST tool, its considerations, and related publications are available on the PROBAST website (www.probast.org). Supplementary file 2A provides a summary table with the criteria used to judge risk of bias.

Two reviewers independently extracted data from each article using the constructed form. To ensure consistent data extraction by all reviewers, we piloted the form on five articles. During the pilot, reviewers clarified differences in interpretation and the standardised data extraction. After the pilot, articles used were randomly assigned and screened again during the main data extraction. One researcher (CLAN) extracted data from all articles, and six researchers (TT, SWJN, PD, JM, RB, and JAAD) collectively extracted data from the same articles. Any disagreements in data extraction were settled by consensus among each pair of reviewers.

### Data analysis

Prediction model studies were categorised as prognosis or diagnosis and subcategorised into four types of prediction models: development (with internal validation), development with external validation (same model), development with external validation (another model), and external validation only.^19^ ^20^

*Model development studies with internal validation*—these studies aim to develop a prediction model to be used for individualised predictions where its predictive performance is directly evaluated using the same data, either by resampling participant data or random or non-random split sample (internal validation).

*Model development studies with external validation of the same model*—these studies have the same aim as the model development studies, but the predictive performance of the model is subsequently quantified in a different dataset.

*Model development studies with external validation of another model*—these studies aim to update or adjust an existing model that performs poorly by recalibrating or extending the model.

*External validation only studies*—these studies aim to assess only the predictive performance of existing prediction models using data external to the development sample.

Two independent reviewers each assessed the signalling questions by the degree of compliance with the PROBAST recommendations. Disagreements were discussed until consensus was reached. The risk of bias judgment for each domain was based on the answers to the signalling questions. If the answer to all signalling questions was yes or probably yes, then the domain was judged as low risk of bias. If reported information was insufficient to answer the signalling questions, these were judged as no information, and the domain was scored as unclear risk of bias. If any signalling question was answered as no or probably no, the reviewers applied their judgment to rate the domain as low, high, or unclear risk of bias.

After judging all the domains, we performed an overall assessment for each application of PROBAST. This tool recommends rating the study as low risk of bias if all domains had low risk of bias. If at least one domain had a high risk of bias, overall judgment should be rated as high risk of bias. If the risk of bias was unclear in at least one domain and all other domains had a low risk of bias then an unclear risk of bias was assigned. The rationale behind judgments was recorded to facilitate discussion among reviewers when solving discrepancies. We removed signalling question 4.9, “Do predictors and their assigned weights in the final model correspond to the results from the reported multivariable analysis?” because it applies to regression based studies. Results were summarised as percentages with corresponding 95% confidence intervals and visual plots. Analyses were performed using R version 3.6.2 (R Core Team, 2020).

### Patient and public involvement

It was not possible to involve participants or the public in setting the research question nor were they involved in the design or implementation of the study or the interpretation or writing up of results. The protocol is available open access https://bmjopen.bmj.com/content/10/11/e038832.

## Results

**Fig 1:**
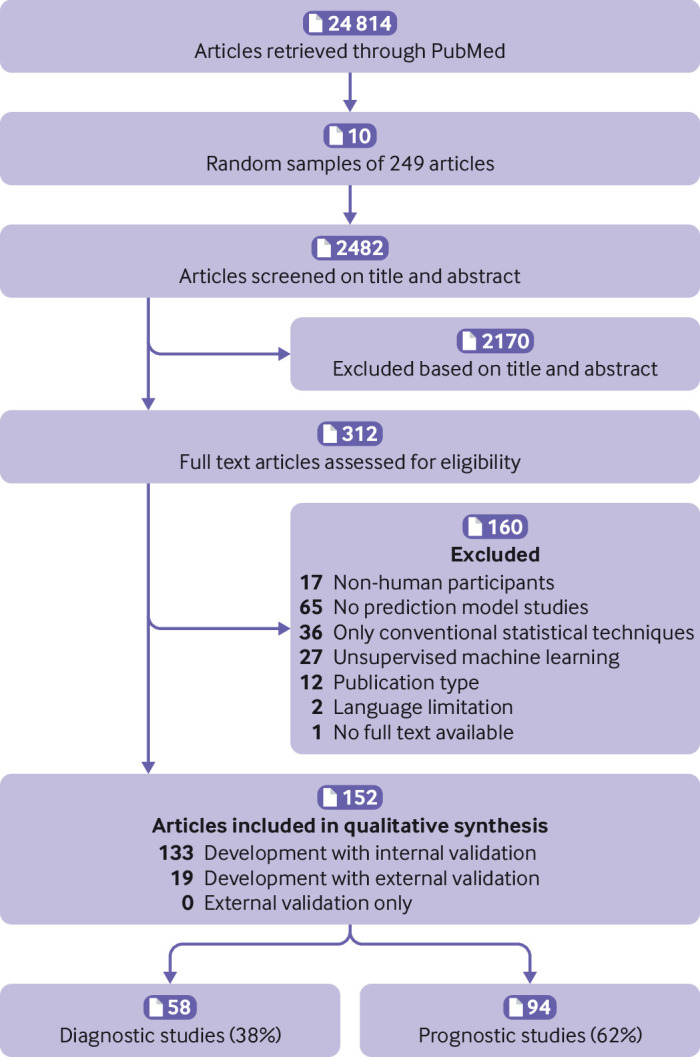
Flowchart of included studies

The search identified 24 814 articles, of which 10 random sets of 249 publications each were sampled. Of the 2482 screened articles, 152 studies were eligible (see supplementary file 3 for details of the studies): 94 (62%) were prognostic and 58 (38%) diagnostic studies of machine learning based prediction models (fig 1). The articles were classified according to the aims of the research: 132 (87%) as model development with internal validation, 19 (13%) as model development with external validation of the same model, and 1 (1%) as model development with external validation of another model (eventually included as development with internal validation). Across the 152 studies, a total of 1429 machine learning based prediction models were developed and 219 validated. For the analyses in the current study, only the model recommended by the authors was selected for the risk of bias assessment. Hence PROBAST was applied 171 times: in 152 developed models and 19 external validations. The most common machine learning techniques for the first model reported were classification and regression tree (10.1%), support vector machine (9.4%), and random forest (9.4%). Supplementary file 3 provides a detailed list of the techniques assessed. The clinical specialties with the most publications were oncology (21/152, 14%), surgery (20/152, 14%), and neurology (20/152, 14%).

### Participants domain

**Fig 2:**
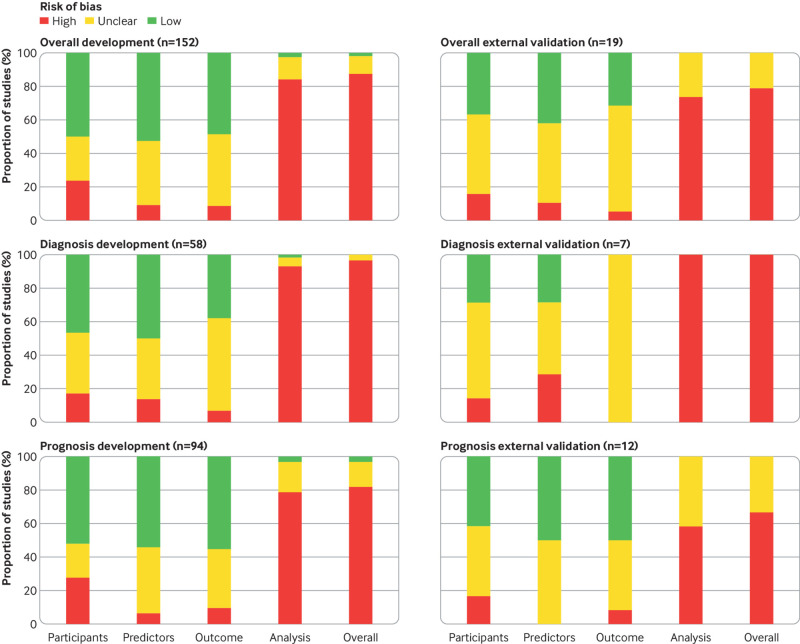
Risk of bias of included studies (n=152) and stratified by study type

In total, 36/152 (24%) developed models and 3/19 (16%) external validations were rated as high risk of bias for the participants domain (fig 2). Prospective and longitudinal data sources (signalling question 1.1) were properly used for model development in 111/152 (73%) and for external validation in 14/19 (74%). It was not possible to evaluate whether the inclusion and exclusion of participants (signalling question 1.2) was representative of the target population in 47/152 (31%) developed models and 12/19 (63%) external validations (table 1).

**Table 1:** PROBAST signalling questions for model development and validation analyses in 152 included studies. Values are number, percentage (95% confidence interval)

Signalling question No	Signalling question	Developed models (n=152)				External validations (n=19)		
		Yes or probably yes	No or probably no	No information		Yes or probably yes	No or probably no	No information
**Participants domain**								
1.1	Were appropriate data sources used, for example, cohort, randomised controlled trial, or nested case-control study data?	111 (73, 66 to 79)	32 (21, 15 to 28)	9 (6, 3 to 11)		14 (74, 51 to 88)	5 (26, 12 to 49)	0
1.2	Were all inclusions and exclusions of participants appropriate?	89 (59, 51 to 66)	16 (11, 7 to 16)	47 (31, 24 to 39)		7 (37, 19 to 59)	0	12 (63, 41 to 81)
**Predictors domain**								
2.1	Were predictors defined and assessed in a similar way for all participants?	109 (72, 64 to 78)	19 (13, 8 to 19)	24 (16, 11 to 22)		8 (42, 23 to 64)	1 (5, 0 to 25)	10 (53, 32 to 73)
2.2	Were predictor assessments made without knowledge of outcome data?	88 (58, 50 to 66)	4 (3, 1 to 7)	60 (40, 32 to 47)		10 (53, 32 to 73)	2 (11, 3 to 31)	7 (37, 19 to 59)
2.3	Were all predictors available at the time the model was intended to be used?	117 (77, 70 to 83)	4 (3, 1 to 7)	31 (20, 15 to 28)		12 (63, 41 to 81)	1 (5, 0 to 25)	6 (32, 15 to 54)
**Outcome domain**								
3.1	Was the outcome determined appropriately?	114 (75, 68 to 81)	6 (4, 2 to 8)	32 (21, 15 to 28)		12 (63, 41 to 81)	0	7 (37, 19 to 6)
3.2	Was a prespecified or standard outcome definition used?	118 (78, 70 to 84)	6 (4, 2 to 8)	28 (18, 13 to 25)		13 (68, 46 to 85)	0	6 (32, 15 to 54)
3.3	Were predictors excluded from the outcome definition?	90 (59, 51 to 67)	8 (5, 3 to 1)	54 (36, 28 to 43)		10 (53, 32 to 73)	0	9 (47, 27 to 69)
3.4	Was the outcome defined and determined in a similar way for all participants?	118 (78, 70 to 84)	11 (7, 4 to 13)	23 (15, 10 to 22)		10 (53, 32 to 73)	1 (5, 0 to 25)	8 (42, 23 to 64)
3.5	Was the outcome determined without knowledge of predictor information?	63 (41, 34 to 49)	10 (7, 4 to 12)	79 (52, 44 to 60)		4 (21, 9 to 43)	1 (5, 0 to 25)	14 (74, 51 to 88)
3.6	Was the time interval between predictor assessment and outcome determination?	110 (72, 65 to 79)	2 (1, 0 to 5)	40 (26, 20 to 34)		11 (60, 36 to 77)	1 (5, 0 to 25)	7 (37, 19 to 59)
**Analysis domain**								
4.1	Were there a reasonable number of participants with the outcome?	52 (34, 27 to 42)	85 (56, 48 to 64)	15 (10, 6 to 16)		8 (42, 23 to 64)	8 (42, 23 to 64)	3 (16, 6 to 38)
4.2	Were continuous and categorical predictors handled appropriately?	37 (24, 18 to 32)	34 (22, 17 to 30)	81 (53, 45 to 61)		0	1 (5, 0 to 25)	18 (95, 75 to 100)
4.3	Were all enrolled participants included in the analysis?	84 (55, 47 to 63)	29 (19., 14 to 26)	39 (26, 19 to 33)		10 (53, 32 to 73)	3 (16, 6 to 38)	6 (32, 15 to 54)
4.4	Were participants with missing data handled appropriately?	20 (13, 9 to 20)	62 (41, 33 to 49)	70 (46, 38 to 54)		3 (16, 6 to 38)	7 (37, 19 to 59)	9 (47, 27 to 68)
4.5	Was selection of predictors based on univariable analysis avoided?	101 (66, 59 to 74)	28 (18, 13 to 25)	23 (15., 10 to 22)		NA		
4.6	Were complexities in the data (e.g., censoring, competing risks, sampling of control participants) accounted for appropriately?	63 (41, 34 to 49)	35 (23, 17 to 30)	54 (36, 28 to 43)		8 (42, 23 to 64)	4 (21, 9 to 43)	7 (37, 19 to 59)
4.7	Were relevant model performance measures evaluated appropriately?	15 (10, 6 to 16)	46 (30, 24 to 38)	91 (60, 52 to 67)		3 (16, 6 to 38)	3 (16, 6 to 38)	13 (68, 46 to 85)
4.8	Were model overfitting and optimism in model performance accounted for?	76 (50, 42 to 58)	59 (39, 31 to 47)	17 (11, 7 to 17)		NA		

Signalling question 4.9 “Do predictors and their assigned weights in the final model correspond to the results from the reported multivariable analysis?” was not included as it applies to regression based studies.

PROBAST=prediction risk of bias assessment tool; NA=not applicable to external validation.

### Predictors domain

Overall, 14/152 (9%) developed models and 2/19 (11%) external validations were rated as high risk of bias for the predictors domain (fig 2). Candidate predictors were defined and assessed in a similar way for all included participants (signalling question 2.1) in 109/152 (72%) developed models and 8/19 (42%) external validations. Information on blinding of predictor assessment to outcome data (signalling question 2.2) was missing in 60/152 (40%) developed models and 7/19 (37%) external validations. All the considered predictors should be available at the time the model is intended to be used (signalling question 2.3), which was considered appropriate in 116/152 (77%) developed models and 12/19 (63%) external validations (table 1).

### Outcome domain

The outcome domain was rated as unclear risk of bias in 65/152 (43%) developed models and 12/19 (63%) external validations (fig 2). Information was missing about the outcome being determined without knowledge of predictors’ information (signalling question Q3.5) in 79/152 (52%) developed models and 14/19 (74%) external validations. Predictors were excluded from the outcome definition (signalling question 3.3) in 90/152 (59%) developed models and 10/19 (53%) external validations. The time interval between predictor measurement and determination of outcome was considered appropriate (signalling question 3.6) in 110/152 (72%) developed models and 11/19 (58%) external validations. In 114/152 (75%) developed models and 12/19 (63%) external validations, the outcome was determined using appropriate methods, thus reducing risk of misclassification (signalling question 3.1). Similarly, 118/152 (78%) developed models and 13/19 (68%) external validations used prespecified, standard, or consensus based definitions to determine the outcome (signalling question 3.2). The outcome was defined and measured with the same categories or thresholds for all included participants (signalling question 3.4) in 118/152 (78%) developed models and 10/19 (53%) external validations (table 1).

### Analysis domain

Overall, 128/152 (84%) developed models and 14/19 (74%) external validations were rated as high risk of bias in the analysis domain. The number of participants with the outcome (signalling question 4.1) was considered insufficient (ie, event per predictor parameter <10) in 85/152 (56%) developed models and 8/19 (42%) external validations (ie, number of events <100). Information about methods to handle continuous and categorical predictors (signalling question 4.2) was missed in 81/152 (53%) developed models and 18/19 (95%) external validations. In total, 84/152 (55%) developed models and 10/19 (53%) external validations included in the statistical analyses all enrolled participants (signalling question 4.3).

Handling of missing data (signalling question 4.4) was inappropriate (ie, participants with missing data were omitted from the analysis or the imputation method was flawed) in 62/152 (41%) developed models and 7/19 (37%) external validations. Overall, 28/152 (18%) developed models used univariable analyses to select predictors (signalling question 4.5). It was not possible to assess if censoring, competing risks, or sampling of control participants (signalling question 4.6) was considered in 54/152 (36%) developed models and 7/19 (37%) external validations. Similarly, the reporting of relevant model performance measures (eg, both discrimination and calibration) (signalling question 4.7) was missing in 91/152 (60%) developed models and 13/19 (68%) external validations. Seventy six (50%) developed models accounted for model overfitting and optimism (signalling question 4.8).

### Overall risk of bias

The overall risk of bias assessed using PROBAST resulted in 133/152 (88%) developed models and 15/19 (79%) external validations being rated as high risk of bias (fig 2). Table 1 shows further information about each signalling question answered as yes or probably yes, no or probably no, and no information.

### Diagnostic versus prognostic models

The analysis domain was the major contributor to an overall high risk of bias in both the diagnostic and the prognostic prediction models. Overall, 56/58 (97%) developed models and 7/7 (100%) external validations were evaluated as high risk of bias in diagnostic studies, and 77/94 (82%) developed models and 8/13 (67%) external validations in prognostic studies (fig 2). External validations of both diagnostic and prognostic models are prone to unclear information to judge risk of bias. While for diagnostic models, signalling questions in the outcome domain were frequently answered with no information (supplementary table S2), for prognostic models this was the case for both the outcome domain and the analysis domain (supplementary table S3). Supplementary file 3 provides further information about each signalling question.

## Discussion

In this study we assessed the methodological quality of studies on supervised machine learning based prediction models across all clinical specialties. Overall, 133/152 (88%) developed models and 15/19 (79%) external validations showed high risk of bias. The analysis domain was most commonly rated as high risk of bias in developed models and external validations, mainly as a result of low number of participants with the outcome (relative to the number of candidate predictors), risk of overfitting, and inappropriate handling of participants with missing data. Although no studies are conclusive about sample size calculations for developing prediction models using machine learning techniques, these studies usually require (many) more participants and events than do conventional statistical approaches.^23^ ^24^ One hundred studies failed to either provide the number of events or report an event per candidate predictor lower than 10, which historically is a marker of potentially low sample size. Furthermore, machine learning studies with a low number of participants with the outcome are prone to overfitting—that is, the model is too much tailored to the development dataset.^23^ ^24^ ^25^ ^26^ Only half of the included studies examined potential overfitting of models by using either split data, bootstrapping, or cross validation. Random split was often relied on to internally validate models (ie, validation based on the same participants’ data), whereas bootstrapping and cross validation are generally considered more appropriate.^27^

Most studies carried out complete case analyses or imputation using means or medians. Multiple imputation is generally preferred as it prevents biased model performance as a result of deletion or single imputation of participants’ missing data. Multiple imputation is, however, still unpopular within models developed with machine learning techniques.^28^ ^29^ Some machine learning techniques have the power to incorporate this missingness by including a separate category of a predictor variable that has missing values.^30^ Therefore, it would be useful if algorithm developers could improve imputation methods and incorporate informative missingness in their models when possible.

Several signalling questions were scored as “no information”, making it impossible for us to judge potential biases. It was often unclear whether all enrolled participants were included in the analyses, how many participants had missing values, and how missing data were handled. Machine learning is a powerful and automated technique that will learn from data; however, if selection bias is present in the dataset, predictions made using the trained machine learning algorithm will also be biased. Similarly, several signalling questions in PROBAST are tailored to identify lack of blinding (signalling questions 2.2, 3.3, and 3.5); however, almost half of included articles failed to report any information for us to assess blinding. Furthermore, model calibration tables or plots were often not presented, whereas classification measures (ie, confusion matrix) were commonly reported with an overreliance on accuracy.^31^ Reporting and assessment of discrimination (the ability to discriminate between cases and non-cases) and calibration (agreement between predictions and observed outcomes) are essential to assess a models’ predictive performance.^31^

### Comparison with other studies

A systematic review of 23 studies about machine learning for diagnostic and prognostic predictions in emergency departments found that analysis was the most poorly rated domain, with 20 studies at high risk of bias.^32^ This study found deficiencies in how continuous variables and missing data were handled, and that model calibration was rarely reported. Another publication about machine learning risk prediction models for triage of patients in the emergency department also considered 22/25 studies at high risk of bias.^33^ A study assessing the performance of diagnostic deep learning algorithms for medical imaging reported 58 of 81 studies classified as overall high risk of bias.^7^ Similar to our results, major deficiencies were found in the analysis domain including the number of events per variable, inclusion of enrolled participants in the analysis, reporting of relevant model performance measures, and overfitting. Recently, a living systematic review about covid-19 prediction models indicated that all 57 studies that used machine learning were at high risk of bias owing to insufficient sample size, unreported calibration, and internal validation based on training-test split.^34^

### Strength and limitations of this study

We evaluated the risk of bias of supervised machine learning based prediction model studies in a broad sample of articles that included prognostic and diagnostic development only and development with external validation studies. After using a validated search strategy, we retrieved nearly 25 000 publications, which is similar to a previous study.^35^ We only screened one 10th of these articles; therefore, our results are presented using confidence intervals to extrapolate them to the whole sample. The present analyses considered results from studies that were published more than one year ago; nevertheless, we expect these findings still to be applicable and relevant for the clinical prediction specialty. We adopted PROBAST as the benchmark for evaluating risk of bias, enhancing the objectivity and consistency; however, this is not without certain limitations. While two signalling question in PROBAST might become less relevant within the machine learning context (ie, selection of predictors based on univariable analysis and reporting of weighted estimates in the final model correspond to the results from the reported multivariable analysis), further signalling questions related to data generation, feature selection, and overfitting might be necessary.

### Implication for researchers, editorial offices, and future research

The number of machine learning based studies is increasing every year; thus, their identification, reporting, and assessment become even more relevant. It will remain a challenge to determine the risk of bias if detailed information about data and modelling approach (including justifications to any decision made that may biases estimates) is not clearly reported in articles. To better judge studies, we recommend that researchers adhere to the transparent reporting of a multivariable prediction model for individual prognosis or diagnosis (TRIPOD) statement.^36^ ^37^ Although TRIPOD was not explicitly developed for machine learning prediction models, all items are applicable. Similarly, while there is yet no risk of bias assessment tool available specifically for supervised machine learning models, we suggest researchers follow PROBAST recommendations to reduce potential biases when planning and modelling primary prediction models using either regression or non-regression models. For example, the adoption of multiple imputation to handle missing values and cross validation or bootstrapping to internally validate the developed models.

Currently, extensions of TRIPOD and PROBAST for prediction models developed using machine learning are under development (TRIPOD-AI, PROBAST-AI).^38^ ^39^ As sample size contributed largely to the overall high risk of bias, future methodological research could focus on determining the appropriate sample sizes for each supervised learning technique. Giving the rapid and constant evolution of machine learning, periodic systematic reviews of prediction model studies need to be conducted. Although high quality machine learning based prediction model studies are scarce, those that stand out need to be validated, recalibrated, and promptly implemented in clinical practice.^34^ To avoid research waste, we suggest that peer reviewers and journal’s editors promote the adherence to reporting guidelines.^5^ Facilitating the documentation of studies (ie, supplementary material, data, and code) and setting an unlimited word count might improve methodological quality assessment, as well as independent validation (ie, replication). Likewise, requesting external validation of prediction models upon submission might help to set minimum standards to ensure generalisability of supervised machine learning based prediction models studies.

### Conclusion

Most studies on prediction models developed using machine learning show poor methodological quality and are at high risk of bias. Factors contributing to the risk of bias include the exclusion of participants, small sample size, poor handling of missing data, and failure to address overfitting. Efforts to improve the design, conduct, reporting, and validation studies of supervised machine learning based prediction models are necessary to boost its application in clinical practice and avoid research waste.

**What is already known on this topic**

Several publications have highlighted the poor methodological quality of regression based prediction model studies
The number of clinical prediction models developed using supervised machine learning is rapidly increasing; however, evidence about methodological quality and risk of bias is scarce

**What this study adds**

Prediction model studies developed using supervised machine learning have poor methodological quality
Limited sample size, poor handling of missing data, and inappropriate evaluation of overfitting contributed largely to the overall high risk of bias
Predictive performance reported on studies may be at high risk of bias, thus caution is needed when interpreting these findings

## Data Availability

The study protocol is available at https://bmjopen.bmj.com/content/10/11/e038832. Detailed extracted data on all included studies are available upon reasonable request to the corresponding author.

## Web extra

Extra material supplied by authors

Supplementary file: search strategy; summary table with criteria to judge risk of bias; table S1 showing characteristics of included studies (n=152); and tables S2 and S3 showing signalling questions for diagnosis and prognosis model studies

## Notes

Contributors: CLAN, JAAD, PD, LH, RDR, GSC, and KGMM conceived and designed the study. CLAN, JAAD, TT, SN, PD, JM, and RB screened the articles and extracted data. CLAN performed data analysis and wrote the first draft of this manuscript. All authors revised the manuscript and approved the final version. CLAN is the guarantor. The corresponding author attests that all listed authors meet authorship criteria and that no others meeting the criteria have been omitted.

The guarantor of this review (CLAN) affirms that the manuscript is an honest, accurate, and transparent account of the study being reported; that no important aspects of the study have been omitted; and that any discrepancies from the study as planned have been explained.

Dissemination to participants and related patient and public communities: We plan to disseminate the findings and conclusions from this study through social media (such as Twitter), a plain language summary on www.probast.org, and scientific conferences. In addition, the findings will provide insights to the development of PROBAST-AI.

Provenance and peer review: Not commissioned; externally peer reviewed.
