# Supplementary material for: Risk of bias in studies on prediction models developed using supervised machine learning techniques: systematic review
Source: BMJ. 2021 Oct 20;375:n2281. doi: 10.1136/bmj.n2281 (PMC8527348; doi:10.1136/bmj.n2281)
Supplement: Supplementary file 1 — Supplementary file: search strategy; summary table with criteria to judge risk of bias; table S1 showing characteristics of included studies (n=152); and tables S2 and S3 showing signalling questions for diagnosis and prognosis model studies [file andc066195.ww.pdf]

Supplementary Material

Risk of bias in studies on prediction models developed using supervised machine learning techniques: systematic review and critical appraisal

Constanza L Andaur Navarro[1,2] *doctoral student* (c.l.andaurnavarro@umcutrecht.nl, 0000-0002-7745-2887), Johanna AA Damen[1,2] *assistant professor* (j.a.a.damen@umcutrecht.nl, 0000-0001-7401-4593), Toshihiko Takada[1] *assistant professor* (t.takada@umcutrecht.nl, 0000-0002-8032-6224), Steven W J Nijman[1] *doctoral student* (S.W.J.Nijman@umcutrecht.nl, 0000-0001-6798-2078), Paula Dhiman[3,4] *research fellow* (paula.dhiman@ndorms.ox.ac.uk, 0000-0002-0989-0623), Jie Ma[3] *medical statistician* (jie.ma@csm.ox.ac.uk, 0000-0002-3900-1903), Gary S Collins[3,4] *professor* (gary.collins@csm.ox.ac.uk, 0000-0002-2772-2316), Ram Bajpai[5] *research fellow* (r.bajpai@keele.ac.uk, 0000-0002-1227-2703), Richard D Riley5 *professor* (r.riley@keele.ac.uk, 0000-0001-8699-0735), Karel GM Moons1,2 *professor* (k.g.m.moons@umcutrecht.nl, 0000-0003-2112-004X), Lotty Hooft1,2 *professor* (l.hooft@umcutrecht.nl,, 0000-0002-7950-2980)

[1]Julius Center for Health Sciences and Primary Care, University Medical Center Utrecht, Utrecht University, Utrecht, The Netherlands.

[2]Cochrane Netherlands, University Medical Center Utrecht, Utrecht University, Utrecht, The Netherlands.

[3]Center for Statistics in Medicine, Nuffield Department of Orthopaedics, Rheumatology & Musculoskeletal Sciences, University of Oxford, Oxford, United Kingdom.

[4]NIHR Oxford Biomedical Research Centre, Oxford University Hospitals NHS Foundation Trust, Oxford, United Kingdom

[5] Centre for Prognosis Research, School of Medicine, Keele University, Keele, United Kingdom.

Supplemental file 1: Search strategy

Search date: 19 November 2019

1. Machine Learning[MeSH Terms]
2. Deep learning[MeSH Terms]
3. supervised machine learning[MeSH Terms]
4. "Neural Networks, Computer"[Mesh]
5. data mining[MeSH Terms]
6. machine[tiab] AND (learn* OR model*)
7. (statistical[tiab] OR "statistical to learning"[tiab]) AND (strateg*[tiab])
8. multilayer perceptron*[tiab] OR random forest*[tiab] OR bayes* network*[tiab] OR support vector machine*[tiab] OR nearest neighbor*[tiab] OR k nearest neighbor*[tiab] OR elastic net[tiab] OR naive bayes*[tiab]
9. (classification[tiab] OR regression[tiab] OR estimation[tiab] OR decision[tiab]) AND tree[tiab]
10. ridge[tiab] OR kernel[tiab] OR ensemble[tiab] OR bagging[tiab] OR bagged[tiab] OR boosting[tiab] OR boosted[tiab] OR fuzzy[tiab]
11. #1 OR #2 OR #3 OR #4 OR #5 OR #6 OR #7 OR #8 OR #9 OR #10
12. (Validat* OR Predict* OR Rule*). [tiab]
13. (Predict* AND (Outcome* OR Risk* OR Model*). [tiab]
14. ((History OR Variable* OR Criteria OR Scor* OR Characteristic* OR Finding* OR Factor*) AND (Predict* OR Model* OR Decision* OR Identif* OR Prognos*)). [tiab]
15. (Decision* AND (Model* OR Clinical*). [tiab]
16. (Prognostic AND (History OR Variable* OR Criteria OR Scor* OR Characteristic* OR Finding* OR Factor* OR Model*). [tiab]
17. #12 OR #13 OR #14 OR #15 OR #16
18. (discrimination[tiab] OR discriminative[tiab] OR discriminatory[tiab]) AND (accuracy[tiab] OR ability[tiab] OR performance[tiab] OR value[tiab] OR model[tiab] OR models[tiab] OR power[tiab] OR capacity[tiab] OR capabilit*[tiab] OR efficiency[tiab])
19. (discriminability[tiab] OR c to index[tiab] OR c to statistic[tiab] OR concordance[tiab] OR DCA[tiab])
20. "decision curve"[tiab]

21. calibrat*[tiab] AND (plot*[tiab] OR curve*[tiab] OR slope*[tiab] OR model[tiab] OR models[tiab])
22. performance[tiab] AND (classification[tiab] OR classifier[tiab] OR clinical[tiab] OR accuracy[tiab] OR validation[tiab] OR metrics[tiab] OR diagnostic[tiab] OR AUC[tiab])
23. (sensitivity[tiab] OR specificity[tiab] OR PPV[tiab] OR NPV[tiab])
24. "correctly classified"[tiab]
25. "clinical accuracy"[tiab]
26. positive predictive value*[tiab]
27. negative predictive value*[tiab]
28. classification[tiab] OR classifier[tiab]
29. Area Under Curve[Mesh]
30. "Area under the curve"[tiab]
31. "Area under the ROC"[tiab]
32. “Area Under the Receiver”[tiab]
33. (ROC[tiab] OR AUC[tiab] OR AUROC[tiab])
34. ROC Curve [Mesh]
35. "Hosmer to Lemeshow"[tiab] OR "H to L test"[tiab]
36. "expected ratio"[tiab] OR "observed ratio"[tiab] OR "E:O ratio"[tiab]
37. #18 OR #19 OR #20 OR #21 OR #22 OR #23 OR #24 OR #25 OR #26 OR #27 OR #28 OR #29 OR #30 OR #31 OR #32 OR #33 OR #34 OR #35 OR #36

---

38. #11 AND #17
39. #11 AND (#17 OR #37)
40. #39 AND (“2018/01/01”[PDat]: “2019/12/31”[PDat])
41. #40 NOT “review”[pt]
42. #39 AND (“2019/01/01”[PDat]: “2019/12/31”[PDat])
43. #42 NOT “review”[pt]

Results #41= **24732**
Results #43=**12977**

Supplemental file 2: Summary table with criteria to judge risk of bias per domain

<table>
<tr><td rowspan="6">PARTICIPANTS</td><td colspan="2">1.1 Were appropriate data sources used, e.g., cohort, RCT or nested case-control study?</td></tr>
<tr><td>↓ Decreases RoB (Y/PY)</td><td>↑ Increases RoB (N/PN)</td></tr>
<tr><td>• Prospective longitudinal cohorts (or proper registry) with consistent methods for inclusion and exclusion of participants, predefined predictors, and outcome determination across a predefined follow-up.
• RCTs with broader inclusion criteria and including treatment as predictor.
• Nested case-control or case-cohort studies adjusted for the original outcome frequency (e.g., inverse sampling fraction)</td><td>• Existing cohorts with potentially inconsistent participant inclusion/exclusion criteria (i.e., data collected for other purposes than developing and validating a prediction model)
• RCTs with narrower eligibility for participants.
• Non-nested case–control design</td></tr>
<tr><td colspan="2">1.2 Were all inclusions and exclusions of participants during enrolment appropriate?</td></tr>
<tr><td>↓ Decreases RoB (Y/PY)</td><td>↑ Increases RoB (N/PN)</td></tr>
<tr><td>• Inclusion/exclusion of participants is appropriate to obtain a representative sample of target population</td><td>• Inappropriate inclusion/exclusion of participants of the target population
• Include participants who have already had the outcome</td></tr>
<tr><td rowspan="10">PREDICTORS</td><td colspan="2">2.1 Were predictors defined and assessed in a similar way for all participants?</td></tr>
<tr><td>↓ Decreases RoB (Y/PY)</td><td>↑ Increases RoB (N/PN)</td></tr>
<tr><td>• Predictors defined and assessed in the same way for all participants</td><td>• Different definitions or assessment of predictors (e.g., pre-op Hb measured using blood test or blood gas)
• Assessment of predictors involved subjective judgement or skilled training which was done by assessors with different experience</td></tr>
<tr><td colspan="2">• Data collected for non-research purposes from multiple sources (i.e., routinely collected data) needs to be scrutinized on the likelihood to have used different definitions.</td></tr>
<tr><td colspan="2">2.2 Were predictor assessments made without knowledge of outcome data?</td></tr>
<tr><td>↓ Decreases RoB (Y/PY)</td><td>↑ Increases RoB (N/PN)</td></tr>
<tr><td>• Outcome information was clearly not (yet) available to those assessing predictors
• Blinding of the outcome</td><td>• Outcome information was used when assessing predictors
• Lack of blinding of the outcome
• Retrospectively recorded predictors</td></tr>
<tr><td colspan="2">2.3 Are all predictors available at the time the model is intended to be used?</td></tr>
<tr><td>↓ Decreases RoB (Y/PY)</td><td>↑ Increases RoB (N/PN)</td></tr>
<tr><td>• Included predictors would be available at the time the model is intended to be applied</td><td>• Included predictors would be unavailable at the time the model is intended to be applied</td></tr>
</table>

		• *For validation studies,* predictor data needed for the model is missing from the validation dataset.
**OUTCOME**	**3.1 Was the outcome determined appropriately?**	
	↓ Decreases RoB (Y/PY)	*↑ Increases RoB (N/PN)*
	• Outcome determination is considered optimal or acceptable by guidelines or previous publications on the topic.	• Suboptimal method to determine outcome, leading to errors in determining the status of participants<br>• Subjective outcomes (e.g., imaging outcomes, outcomes at surgeon discretion, or ones which need special skill training)
	**3.2 Was a prespecified or standard outcome definition used?**	
	↓ Decreases RoB (Y/PY)	*↑ Increases RoB (N/PN)*
	• Prespecified or standard outcome definition is used and substantiated by a definition from clinical guidelines, previously published studies, or a published study protocol	• Atypical threshold on a continuous scale has been used<br>• Composite outcomes that exclude atypical components<br>• Consensus-based outcomes
	**3.3 Were predictors excluded from the outcome definition?**	
	↓ Decreases RoB (Y/PY)	*↑ Increases RoB (N/PN)*
	• Outcome determined without using predictors information	• Any predictor forms part of the outcome definition<br>• Outcome determined using consensus panel
	**3.4 Was the outcome defined and determined in a similar way for all participants**	
	↓ Decreases RoB (Y/PY)	*↑ Increases RoB (N/PN)*
	• Outcome was defined and determined in a similar way for all participants	• Outcome was clearly defined and determined in different way for some participants
	**3.5 Was the outcome determined without knowledge of predictor information?**	
	↓ Decreases RoB (Y/PY)	*↑ Increases RoB (N/PN)*
	• Information about predictors is not known when determining the outcome status<br>• Studies clearly reported outcome status was determined without knowledge of predictor information<br>• Objective outcome	• Information about predictors is used to determine the outcome status
	**3.6 Was the time interval between predictor assessment and outcome determination appropriate?**	
	↓ Decreases RoB (Y/PY)	*↑ Increases RoB (N/PN)*

<table>
<tr><td></td><td>• Time interval between predictor assessment and outcome determination enables to correctly record the outcome and achieve a representative number of events.</td><td>• Time interval between predictor assessment and outcome determination is either too long or too short to correctly record the outcome and achieve a representative number of events.</td></tr>
<tr><td rowspan="12">ANALYSIS</td><td colspan="2">4.1 Were there a reasonable number of participants with the outcome?</td></tr>
<tr><td>↓ Decreases RoB (Y/PY)</td><td>↑ Increases RoB (N/PN)</td></tr>
<tr><td>• For model development studies, if the number of participants with the outcome relative to the number of candidate predictor parameters (EPV) is ≥20
• For model validation studies, if the number of participants with the outcome is ≥100</td><td>• For model development studies, if the number of participants with the outcome relative to the number of candidate predictor parameters is <10
• For model validation studies, if the number of participants with the outcome is <100</td></tr>
<tr><td colspan="2">4.2 Were continuous and categorical handled appropriately?</td></tr>
<tr><td>↓ Decreases RoB (Y/PY)</td><td>↑ Increases RoB (N/PN)</td></tr>
<tr><td>• Continuous predictors are not dichotomized
• Continuous predictors are categorized based on clinical cut-points
• Continuous predictors are examined for nonlinearity
• For validation studies, predictors are collected using same definitions or categorized using same cut-points</td><td>• Continuous predictors are dichotomised
• Continuous predictors are categorised using widely accepted clinical cut-points or data driven cut-points
• For validation studies, predictors are collected using different definitions or categorized using different cut-points</td></tr>
<tr><td colspan="2">4.3 Were enrolled participants included in the analysis?</td></tr>
<tr><td>↓ Decreases RoB (Y/PY)</td><td>↑ Increases RoB (N/PN)</td></tr>
<tr><td>• All participants who met the inclusion criteria in the study are included in the analysis, or a very low number are excluded</td><td>• Some participants or subgroups are inappropriately excluded from the analysis (e.g., participants with ‘unclear’ findings, missing data, outliers, incomplete follow-up)</td></tr>
<tr><td colspan="2">4.4 Were participants with missing data handled appropriately?</td></tr>
<tr><td>↓ Decreases RoB (Y/PY)</td><td>↑ Increases RoB (N/PN)</td></tr>
<tr><td>• No missing values of predictors or outcomes. The study explicitly reports that participants are not excluded based on missing data
• Missing data are handled using multiple imputation
• Comparing results with and without missing data</td><td>• Missing data are omitted from the analysis (e.g., complete-case analysis)
• Method for handling missing data is clearly flawed (e.g., missing indicator method or inappropriate use of last value carried forward)
• Study had no explicit mention of methods to handle missing data</td></tr>
</table>

	• *For validation studies,* omitting systematically missing predictors.	
	**4.5 Was selection of predictors based on univariable analysis avoided? †**	
	↓ *Decreases RoB (Y/PY)*	↑ *Increases RoB (N/PN)*
	• Predictors selected on existing knowledge and forced into the model<br>• Any methods not based on prior statistical test between predictor and outcome (e.g., principal component analysis)<br>• Multivariable selection strategy during modelling needs to be testing for overfitting	• Predictors are selected based on univariable analysis prior to multivariable modelling
	**4.6 Were complexities in the data (e.g., censoring, competing risks, sampling of control participants) accounted for appropriately?**	
	↓ *Decreases RoB (Y/PY)*	↑ *Increases RoB (N/PN)*
	• Case-cohort or nested case-control design account for sampling fractions.<br>• Cox regression is used for long-term outcomes in which censoring occurs.<br>• Multilevel or random effects models for multiple events for the same outcome	• Complexities in the data are not accounted for appropriately<br>• Competing risk are ignored
	**4.7 Were relevant model performance measures evaluated appropriately?**	
	↓ *Decreases RoB (Y/PY)*	↑ *Increases RoB (N/PN)*
	• Both calibration and discrimination are evaluated appropriately<br>• Performance measures accounting for censoring are used in models predicting survival outcomes (e.g., D-statistics, Harrell's c-index)	• Both calibration and discrimination are not evaluated<br>• Only goodness-of-fit tests are used to evaluate calibration (e.g., Hosmer–Lemeshow)<br>• Performance measures accounting for censoring are not used in models predicting survival outcomes<br>• Classification measures (e.g., sensitivity, specificity, or predictive values) were presented using predicted probability thresholds derived from the data set at hand or based on non-clinical cut-points
	**4.8 Was model overfitting and optimism in model performance accounted for?†**	
	↓ *Decreases RoB (Y/PY)*	↑ *Increases RoB (N/PN)*
	• Internal validation using bootstrapping or cross-validation, and subsequent adjustment of the model performance estimates have been applied, if necessary.	• No internal validation has been performed, or if internal validation consists only of a single random split-sample of participant data

		• Bootstrapping or cross-validation did not include all model development procedures (e.g., variable selection procedure)

We removed signalling question 4.9 -Do predictors and their assigned weights in the final model correspond to the results from the reported multivariable analysis?
Unclear RoB: if relevant information is missing for some of the signalling questions and all other signalling questions in the domain were answer as Y/PY.
† Signalling questions applicable only to development studies
Criteria are based on PROBAST tool. For details, please visit www.probast.org
Abbreviations: RoB, risk of bias; Y, yes; PY, probably yes; N, no; PN, probably no; EPV, events per variable.

Supplemental file 3: Characteristics of included studies

Table S1. Characteristics of included studies (n=152)

First Author	Journal	Impact factor [a]	Publication year	Clinical field	Outcome	Study design
**Prognosis**						
X Jiang(1)	PLoS ONE	2.740	2019	Oncology	5-year breast cancer metastasis	Development with external validation (same model)
L Adhikari(2)	PLoS ONE	2.740	2019	Nephrology	Acute kidney injury at first 7 days after surgery	Development only (including internal validation)
G Lorenzoni(3)	Journal of Clinical Medicine	3.303	2019	Cardiovascular medicine	First hospitalization in heart failure patients	Development only (including internal validation)
L-K Pries(4)	Schizophrenia Bulletin	7.958	2019	Psychiatry	Schizophrenia	Development with external validation (same model)
I Sánchez Fernández(5)	Journal of Child Neurology	2.092	2018	Neurology	In-hospital mortality in critically ill children monitored with cEEG in the ICU	Development only (including internal validation)
GGP Garcia(6)	American Journal of Ophthalmology	4.483	2018	Ophthalmology	progression normal tension glaucoma	Development only (including internal validation)
A Tam(7)	GigaScience	5.993	2019	Neurology	Progression to Alzheimer's dementia	Development with external validation (same model)
V Bhat(8)	Mayo Clinic Proceeding	7.091	2018	Surgery	New-onset diabetes after transplant	Development only (including internal validation)
KG Friedman(9)	Ultrasound in Obstetrics & Gynecology	5.595	2018	Neonatology	Circulation type	Development only (including internal validation)
H Duan(10)	BMC Medical Informatics and Decision Making	2.317	2019	Cardiovascular medicine	Major adverse cardiac event	Development only (including internal validation)
J Kwon(11)	Resuscitation	4.215	2019	Cardiovascular medicine	neurological recovery after ROSC	Development only (including internal validation)
R Hammond(12)	PLoS ONE	2.740	2019	Nutrition	Obesity status at the age of five	Development only (including internal validation)
AL Nobles(13)	Proceedings of the SIGCHI Conference on Human Factor in Computing Systems([c])	-	2018	Psychiatry	Suicidality	Development only (including internal validation)

NW Sterling(14)	International Journal of Medical Informatics	3.025	2019	Emergency medicine	ED disposition	Development only (including internal validation)
FB Bouallegue(15)	Journal of Alzheimer's Disease	3.517	2018	Neurology	Alzheimer's disease	Development only (including internal validation)
T-L Tsai(16)	Journal of Clinical Medicine	3.303	2019	Critical care	Successful extubation	Development only (including internal validation)
RR Lopes(17)	Netherlands Heart Journal	1.933	2019	Cardiovascular medicine	Mortality	Development only (including internal validation)
G Maragatham(18)	Journal of Medical Systems	3.058	2019	Cardiovascular medicine	Heart failure	Development only (including internal validation)
C-Y Shao(19)	Thoracic Cancer	2.610	2019	Surgery	anastomosis leakage after esophagectomy	Development only (including internal validation)
M Cearns(20)	Translational Psychiatry	5.280	2019	Psychiatry	Re-hospitalization within 2 years of major depressive episode	Development only (including internal validation)
NB Huben(21)	Journal of Endourology	2.267	2018	Urology	Operative time for RARP	Development only (including internal validation)
AT Hale(22)	Neurosurgical focus	2.891	2018	Critical care	Death or alive with GOS score ≤ 3	Development only (including internal validation)
C Salvatore(23)	Journal of Neuroscience Methods	2.785	2018	Neurology	Cognitive status (HC; ncMCI; cMCI; AD)	Development only (including internal validation)
M Zhou(24)	BMC Medical Informatics and Decision Making	2.317	2019	Preventive care	Exercise relapse	Development only (including internal validation)
X Kang(25)	Journal of Maternal-Fetal & Neonatal Medicine	1.737	2019	Obstetrics & Gynecology	Gestational diabetes mellitus with macrosomia	Development only (including internal validation)
CM Sauer(26)	PLoS ONE	2.776	2018	Infectious diseases	Tuberculosis treatment failure	Development only (including internal validation)
LW Thornblade(27)	Journal for Electronic Health data and Methods([c])	-	2018	Surgery	Elective colon resection	Development only (including internal validation)
VJ Lei(28)	Studies in Health Technology and Informatics	0.71[b]	2019	Surgery	All-cause in-hospital mortality	Development only (including internal validation)
SJ Lee(29)	Studies in Health Technology and Informatics	0.71[b]	2019	Oncology	Cancer recurrence	Development only (including internal validation)
GB Auffenberg(30)	European Urology	18.728	2019	Urology	Prostate cancer treatment option	Development only (including internal validation)
Z Wang(31)	Journal of Biomedical Informatics	3.526	2019	Cardiovascular medicine	1-year mortality	Development only (including internal validation)

A Nelson(32)	np Digital Medicine	0.00	2019	Healthcare services	Schedule appointment attendance	Development only (including internal validation)
T Shibahara(33)	JCO Clinical Cancer Informatics([b])	0.43	2018	Oncology	Blood cell count	Development only (including internal validation)
R Chen(34)	Circulation-Cardiovascular Quality and Outcomes	5.071	2019	Cardiovascular medicine	Heart failure	Development only (including internal validation)
Y Fan(35)	Endocrine	3.235	2019	Surgery	Tumor remission after transphenoidal surgery (TSS)	Development only (including internal validation)
A Ferre(36)	Journal of Clinical Sleep Medicine	3.586	2019	Neurology	RDI equal to or above 10 events/h	Development with external validation (same model)
F Zhang(37)	Metabolomics	3.167	2018	Oncology	Recurrence of Epithelial Ovarian Cancer at 5-years	Development only (including internal validation)
AHS Harris(38)	The Journal of Arthroplasty	3.524	2018	Surgery	30-day mortality	Development only (including internal validation)
KM Kuo(39)	BMC Medical Informatics and Decision Making	2.317	2019	Psychiatry	Hospital-acquired pneumonia	Development only (including internal validation)
Y Arai(40)	Blood advances	4.910	2019	Immunology	Acute graft-versus-host disease	Development only (including internal validation)
L Liu(41)	BMC Systems Biology	2.048	2018	Traumatology	Side effects of analgesics	Development only (including internal validation)
C Shappell(42)	Critical Care Medicine	6.971	2018	Critical care	In-hospital mortality	Development only (including internal validation)
X Niu(43)	Scientific Reports	4.011	2018	Cardiovascular medicine	MACEs within 1-year follow-up	Development only (including internal validation)
F Ge(44)	Journal of Affective Disorders	3.892	2019	Psychiatry	Posttraumatic stress disorder at 3 months	Development only (including internal validation)
B Rohaut(45)	Scientific Reports	3.998	2019	Medical imaging	Consciousness at ICU discharge	Development only (including internal validation)
JM Karnuta(46)	The Journal of Arthroplasty	3.524	2019	Surgery	Inpatient payments prior to lower extremity arthroplasty	Development only (including internal validation)
S Cohen(47)	Autism Research	3.697	2018	Psychiatry	Daytime challenging behaviors	Development only (including internal validation)
SHA Faruqui(48)	JMIR MHealth and UHealth	4.313	2019	Endocrinology	Blood glucose level for type 2 Diabetes Mellitus	Development only (including internal validation)
M Molinari(49)	Transplantation	4.546	2019	Surgery	90-day mortality	Development only (including internal validation)

VE Staartjes(50)	Neurosurgical Focus	2.891	2018	Surgery	Gross-total resection in transsphenoidal surgery for pituitary adenoma at 3 months	Development only (including internal validation)
N Park(51)	PLoS ONE	2.776	2018	Oncology	AKI occurrence in 14 days	Development only (including internal validation)
D Chen(52)	Clinical Cancer Informatics	0.43)	2019	Oncology	Time to first treatment in Chronic Lymphocytic Leukemia	Development only (including internal validation)
J Malycha(53)	Resuscitation	4.215	2019	Critical care	FiO2 Added value	Development only (including internal validation)
Z Xie(54)	Preventing chronic disease	2.144	2019	Endocrinology	type 2 diabetes risk	Development only (including internal validation)
A Rozet(55)	Journal of Medical Internet Research	5.034	2019	Psychiatry	Self-reported stress over 100 days	Development only (including internal validation)
AV Karhade(56)	The Spine Journal	3.191	2019	Surgery	Prolonged opioid prescription after surgery for lumbar disc herniation to at least 90 to 180 days postoperatively	Development only (including internal validation)
M Mulder(57)	Archives of Physical Medicine and Rehabilitation	3.098	2019	Neurology	Community walkers after stoke	Development only (including internal validation)
J Debedat(58)	Diabetes Care	15.270	2018	Endocrinology	Type 2 diabetes relapse after Gastric Bypass	Development with external validation (same model)
JCR Alcantud(59)	PLoS ONE	2.740	2019	Oncology	5-years survival rate	Development only (including internal validation)
A Sandstrom(60)	PLoS ONE	2.740	2019	Obstetrics & Gynecology	Preeclampsia with delivery <34 weeks of gestation	Development only (including internal validation)
JN Cooper(61)	Journal of surgical research	1.872	2018	Surgery	30-day postoperative neonatal mortality	Development with external validation (same model)
C-S Rau(62)	PLoS ONE	2.776	2018	Surgery	In-hospital mortality after severe traumatic brain injury	Development only (including internal validation)
RS Anand(63)	AMIA Joint Summits on Translational Sciences Proceedings[c]	-	2018	Critical care	All cause in-hospital mortality	Development only (including internal validation)
Y Aperstein(64)	PLoS ONE	2.740	2019	Critical care	ICU mortality	Development only (including internal validation)
J Park(65)	Journal of Medical Internet Research	5.034	2019	Cardiovascular medicine	Cardio-cerebrovascular event in patients with hypertension	Development with external validation (same model)

R Gupta(66)	Canadian Journal of Ophthalmology	1.369	2019	Ophthalmology	Visual outcome after open globe injury	Development only (including internal validation)
A Kilic(67)	Annals of Thoracic Surgery	3.639	2019	Surgery	Operative mortality	Development only (including internal validation)
C Campillo-Artero(68)	PLoS One	2.776	2018	Obstetrics & Gynaecology	Emergency cesarean section	Development only (including internal validation)
WS Hong(69)	PLoS One	2.776	2018	Healthcare services	patient's disposition (discharge, admission)	Development only (including internal validation)
O Beauchet(70)	Journal of Nutrition Health and Aging	2.660	2018	Geriatric	Fall in acute care medical wards	Development only (including internal validation)
Z Ma(71)	PLoS One	2.776	2018	Cardiovascular medicine	Warfarin dose	Development only (including internal validation)
SPK Veeranki(72)	Studies in Health Technology and Informatics	0.71[b]	2019	Neurology	Delirium	Development only (including internal validation)
H Maharlou(73)	Healthcare Informatics Research	2.939	2018	Healthcare services	Length of stay in ICU after cardiac surgery	Development only (including internal validation)
A Talaei-Khoei(74)	International Journal of Medical Informatics	2.731	2018	Endocrinology	Type 2 diabetes risk at 1, 3 and 8 years	Development only (including internal validation)
D Bertsimas(75)	Annals of Surgery	9.476	2018	Surgery	30-day mortality	Development with external validation (same model)
C Liu(76)	Abdominal Radiology	2.429	2019	Oncology	Lymphadenectomy extension in gastric cancer before surgical resection	Development only (including internal validation)
G Luo(77)	JMIR Medical Informatics	2.577	2019	Healthcare services	Appropriate hospital admission for patients with bronchiolitis	Development with external validation (same model)
M TakeuchI(78)	Journal of Gastrointestinal Surgery	2.686	2018	Oncology	Post-operative overall survival and disease-free survival	Development only (including internal validation)
H Kiiski(79)	Brain Topography	3.104	2018	Neurology	Cognitive functioning and processing speed over 2-year	Development only (including internal validation)
Z Hasnain(80)	PLoS ONE	2.740	2019	Oncology	Post-cystectomy recurrence	Development only (including internal validation)
J Dean(81)	Clinical and Translational Radiation Oncology	1.439	2018	Oncology	Severe acute dysphagia resulting from head and neck radiotherapy	Development with external validation (same model)
M Cheung(82)	Surgery	3.476	2018	Surgery	Mortality in burn patients	Development only (including internal validation)

JL Gowin(83)	NeuroImage: Clinical	4.350	2019	Psychiatry	Relapse rate at 12 months after treatment	Development with external validation (same model)
JC Rojas(84)	Annals of the American Thoracic Society	4.026	2018	Healthcare services	Intensive care unit readmission	Development with external validation (same model)
J Balani(85)	Obstetric Medicine	0.389[b]	2018	Endocrinology	Gestational diabetes mellitus	Development only (including internal validation)
L Gao(86)	Journal of Neurotrauma	4.056	2019	Critical care	Mortality after severe traumatic brain injury at 6 months	Development only (including internal validation)
A Garcia-Arce(87)	Journal for Healthcare Quality	1.092	2018	Healthcare services	Preventable readmission within 30-days	Development only (including internal validation)
CV Cosgriff(88)	npj Digital Medicine([c])	-	2019	Critical care	Illness severity score	Development only (including internal validation)
J Lotsch(89)	Breast Cancer Research and Treatment	3.471	2018	Oncology	Persistent pain after breast cancer surgery at 3 years	Development only (including internal validation)
II Spyroglou(90)	BMC Research Notes	1.38[b]	2018	Immunology	Asthma exacerbation	Development only (including internal validation)
A Facciorusso(91)	Pancreatology	3.629	2019	Oncology	Pain response to repeat echoendoscopic celiac plexus neurolysis	Development only (including internal validation)
DW Kim(92)	Bone	4.360	2018	Dentistry	Occurrence of BRONJ associated with dental extraction	Development only (including internal validation)
AV Karhade(93)	Spine Journal	3.191	2019	Surgery	In-hospital and 90-day post-discharge mortality in SEA	Development only (including internal validation)
T van Steenkiste(94)	Artificial Intelligence in Medicine	4.383	2019	Critical care	Positive blood culture at 72hr	Development only (including internal validation)
N Paliwal(95)	Neurosurgical Focus	2.891	2018	Surgery	Diverters treatment outcome (Occlusion vs. residual)	Development only (including internal validation)
**Diagnosis**						
WP Chen(96)	BioMed Research International	2.276	2018	Dentistry	Periodontitis	Development only (including internal validation)
H Zhang(97)	GigaScience	4.688	2018	Neurology	Alzheimer’s disease	Development with external validation (same model)
A Koivu(98)	Computers in Biology and Medicine	2.286	2018	Obstetrics & Gynecology	First trimester prenatal down's syndrome	Development with external validation (same model)

K Kajiwara(99)	Journal of Vascular and Interventional Radiology	2.828	2018	Oncology	Insulinomas	Development only (including internal validation)
LC Chambers(100)	Sexually Transmitted Diseases	2.270	2018	Healthcare services	Need for a standard visit	Development only (including internal validation)
H Won Choi(101)	American Journal of Roentgenology	3.161	2018	Medical imaging	Early prediction of the severity of acute pancreatitis	Development only (including internal validation)
A Ogunleye(102)	IEEE/ACM Transactions on Computational Biology and Bioinformatics	3.015[b]	2019	Nephrology	Chronic kidney disease	Development only (including internal validation)
CC Wu(103)	Computer Methods and Programs in Biomedicine	3.424	2018	Hepatology	Early fatty liver disease	Development only (including internal validation)
D Shigemi(104)	The Journal of Maternal-Fetal & Neonatal Medicine	1.737	2019	Obstetrics & Gynecology	Macrosomia	Development only (including internal validation)
M Ansart(105)	Statistical Methods in Medical Research	2.291	2019	Neurology	Brain amyloidosis	Development with external validation (same model)
S Zamboni(106)	World Journal of Urology	3.217	2019	Oncology	Adverse pathologic features	Development only (including internal validation)
R Tse(107)	American Journal of Forensic Medicine and Pathology	0.539	2018	Forensic pathology	Salt water drowning with immersion time of less than 1 hour (SWD1)	Development only (including internal validation)
S Perveen(108)	Scientific Reports	4.011	2018	Hepatology	Non-alcoholic fatty liver disease risk	Development only (including internal validation)
Z Pei(109)	Interdisciplinary Sciences-Computational life Sciences	1.418	2018	Primary care	Essential hypertension	Development only (including internal validation)
V Sacca(110)	Brain Imaging and Behavior	3.418	2018	Neurology	Early multiple sclerosis	Development only (including internal validation)
MJRJ Bouts(111)	Human Brain Mapping	4.421	2019	Neurology	Mild cognitive impairment	Development with external validation (same model)
H Yang(112)	IEEE Journal of Biomedical and Health Informatics	5.223	2019	Neurology	Dementia	Development only (including internal validation)
S Liang(113)	Schizophrenia Research	4.569	2018	Psychiatry	Schizophrenia/depression/ Healthy/controls	Development only (including internal validation)
E Klang(114)	Neuroradiology	2.238	2019	Medical imaging	Use of non-contrast CT in ED department	Development only (including internal validation)
R Ferrer-Peña(115)	Journal of Manipulative and Physiological Therapeutics	1.230	2019	Physical medicine	Needle length	Development only (including internal validation)

JM Cameron(116)	Analyst	3.978	2019	Oncology	Brain tumor	Development only (including internal validation)
JK Paul(117)	Computers in Biology and Medicine	3.434	2019	Neurology	Fibromyalgia	Development only (including internal validation)
B Dhondt(118)	World Journal of Urology	3.217	2019	Oncology	Pligometastic vs polymetastatic in prostatic cancer	Development only (including internal validation)
MB Wilson(119)	Otolaryngology-Head and Neck Surgery	2.341	2019	Otolaryngology	Peritonsillar abscess	Development only (including internal validation)
B Lu(120)	Sensors	3.275	2019	Oncology	Lung cancer	Development only (including internal validation)
Y Xu(121)	BMC Cancer	3.150	2019	Oncology	Breast cancer recurrence	Development only (including internal validation)
Y Wang(122)	Academic Radiology	2.488	2019	Medical imaging	Differentiation between T2 and T3/T4 stage in gastric cancer	Development only (including internal validation)
A Mortezagholi(123)	Asian pacific journal of cancer prevention	0.00	2019	Oncology	Gastric cancer	Development only (including internal validation)
UJ Muehlematter(124)	European Radiology	3.962	2018	Medical imaging	Vertebral insufficiency fractures	Development only (including internal validation)
JP Jeon(125)	Clinical Neurology and Neurosurgery	1.672	2018	Surgery	Persistent hemodynamic depression following CAS	Development only (including internal validation)
C-F Lu(126)	Clinical Cancer Research	8.911	2018	Medical imaging	Glioblastoma vs lower grade gliomas	Development with external validation (same model)
T Ballarini(127)	NeuroImage: Clinical 21	4.350	2019	Neurology	Individual treatment response	Development only (including internal validation)
B Thanathornwong(128)	Health Informatics Research	2.939	2018	Dentistry	Need of orthodontic treatment in permanent dentition	Development only (including internal validation)
CQ Ngo(129)	Annual International Conference of the IEEE Engineering in Medicine and Biology Society	1.01[b]	2018	Endocrinology	Hypoglycemia episode	Development only (including internal validation)
SH Hyun(130)	Clinical Nuclear Medicine	6.622	2019	Oncology	adenocarcinoma vs squamous cell carcinoma	Development only (including internal validation)
MS Mellem(131)	Biological Psychiatry: CNNI	5.335	2019	Psychiatry	Transdiagnostic Symptom Severity	Development only (including internal validation)
F Zhang(132)	Neuroscience	5.679	2019	Neurology	Alzheimer's disease	Development only (including internal validation)

D Leightley(133)	Journal of Mental Health	2.604	2018	Psychiatry	Post-traumatic stress disorder	Development only (including internal validation)
A Sandstrom(60)	PLoS ONE	2.740	2019	Obstetrics & Gynecology	Preeclampsia with delivery <34 weeks of gestation	Development only (including internal validation)
C Xiao(134)	Annual International Conference of the IEEE Engineering in Medicine and Biology Society	1.01[b]	2018	Neurology	Parkinson's disease	Development only (including internal validation)
D Gökçay(135)	IEEE Journal of Biomedical and Health Informatics	5.223	2019	Rheumatology	Fibromyalgia	Development only (including internal validation)
AH Butt(136)	BioMedical Engineering OnLine	2.013	2018	Neurology	Patients with Parkinson disease	Development only (including internal validation)
HS Hunter-Zinck(137)	Journal of the American Medical Informatics Association	4.112	2019	Healthcare services	Emergency department orders	Development only (including internal validation)
ML Zhang(138)	American Journal of Clinical Pathology	2.094	2019	Pathology	PBFC with current/recent CBC/differential	Development only (including internal validation)
C Castillo-Olea(139)	International Journal of Environmental Research and Public Health	2.468	2019	Geriatric	Sarcopenia	Development only (including internal validation)
C Sa-ngamuang(140)	PLoS neglected tropical diseases	4.487	2018	Infectious diseases	Dengue	Development only (including internal validation)
K Meena(141)	Artificial Intelligence in Medicine	4.383	2019	Pediatrics	Anemia status in children	Development only (including internal validation)
R Wei(142)	Technology in Cancer Research & Treatment	2.074	2019	Medical imaging	Pre-operative serous cystic neoplasms	Development with external validation (same model)
S Papini(143)	Journal of Anxiety Disorders	3.472	2018	Psychiatry	Posttraumatic stress disorders screening status 3 months post hospitalization	Development only (including internal validation)
B-S Jang(144)	Scientific Reports	4.011	2018	Medical imaging	Pseudoprogression in patients with glioblastoma	Development with external validation (same model)
H Kim(145)	JMIR MHealth and UHealth	4.313	2019	Psychiatry	Depression	Development only (including internal validation)
B Goudey(146)	Scientific Reports	3.998	2019	Neurology	Abnormal CSF Aβ1-42 level	Development only (including internal validation)
W Tu(147)	Journal of NeuroVirology	2.354	2019	Neurology	HIV-associated neurocognitive disorder	Development only (including internal validation)

M Bronsert(148)	American Journal of Surgery	2.125	2019	Surgery	Postoperative complications	Development only (including internal validation)
A Eill(149)	Brain Connectivity	5.263	2019	Neurology	Autism spectrum disorders	Development only (including internal validation)
F Cook(150)	British Journal of Anaesthesia	6.880	2019	Surgery	Intubation difficulty	Development only (including internal validation)
B Eggleston(151)	Brain Injury	1.690	2019	Healthcare services	Service-connected disability (SCD) ≥50 among a cohort of veterans with previous combat deployment	Development only (including internal validation)
YR Villarreal(152)	Social Work in Public Health	0.607	2019	Primary care	Hepatitis C Virus Incidence	Development only (including internal validation)

[a] Value is based on the Journal Citation Report from the year of publication of the article.
[b] Value is based on the Scientific Journal Ranking from the year of publication of the article.
[c] Value is unavailable.

References list of included studies

1. Jiang X, Wells A, Brufsky A, Neapolitan R. A clinical decision support system learned from data to personalize treatment recommendations towards preventing breast cancer metastasis. *PLoS One*. 2019;14(3):1-18. doi:10.1371/journal.pone.0213292

2. Adhikari L, Ozrazgat-Baslanti T, Ruppert M, et al. Improved predictive models for acute kidney injury with IDEA: Intraoperative data embedded analytics. *PLoS One*. 2019;14(4):1-26. doi:10.1371/journal.pone.0214904

3. Chen WP, Chang SH, Tang CY, Liou ML, Tsai SJJ, Lin YL. Composition Analysis and Feature Selection of the Oral Microbiota Associated with Periodontal Disease. *Biomed Res Int*. 2018;2018. doi:10.1155/2018/3130607

4. Lorenzoni G, Sabato SS, Lanera C, et al. Comparison of Machine Learning Techniques for Prediction of Hospitalization in Heart Failure Patients. *J Clin Med*. 2019;8(9):1298. doi:10.3390/jcm8091298

5. Pries LK, Lage-Castellanos A, Delespaul P, et al. Estimating Exposome Score for Schizophrenia Using Predictive Modeling Approach in Two Independent Samples: The Results from the EUGEI Study. *Schizophr Bull*. 2019;45(5):960-965. doi:10.1093/schbul/sbz054

6. Sánchez Fernández I, Sansevere AJ, Gaínza-Lein M, Kapur K, Loddenkemper T. Machine Learning for Outcome Prediction in Electroencephalograph (EEG)-Monitored Children in the Intensive Care Unit. *J Child Neurol*. 2018;33(8):546-553. doi:10.1177/0883073818773230

7. Zhang H, Zhu F, Dodge HH, Higgins GA, Omenn GS, Guan Y. A similarity-based approach to leverage multi-cohort medical data on the diagnosis and prognosis of Alzheimer's disease. *Gigascience*. 2018;7(7):1-10. doi:10.1093/gigascience/giy085

8. Koivu A, Korpimäki T, Kivelä P, Pahikkala T, Sairanen M. Evaluation of machine learning algorithms for improved risk assessment for Down's syndrome. *Comput Biol Med*. 2018;98(April):1-7. doi:10.1016/j.compbiomed.2018.05.004

9. Garcia GGP, Nitta K, Lavieri MS, et al. Using Kalman Filtering to Forecast Disease Trajectory for Patients With Normal Tension Glaucoma. *Am J Ophthalmol*. 2019;199:111-119. doi:10.1016/j.ajo.2018.10.012

10. Kajiwara K, Yamagami T, Toyota N, et al. New Diagnostic Criteria for the Localization of Insulinomas with the Selective Arterial Calcium Injection Test: Decision Tree Analysis. *J Vasc Interv Radiol*. 2018;29(12):1749-1753. doi:10.1016/j.jvir.2018.05.015

11. Tam A, Dansereau C, Iturria-Medina Y, et al. A highly predictive signature of cognition and brain atrophy for progression to Alzheimer's dementia. *Gigascience*. 2019;8(5):1-16. doi:10.1093/gigascience/giz055

12. Chambers LC, Manhart LE, Katz DA, et al. Comparison of Algorithms to Triage Patients to Express Care in a Sexually Transmitted Disease Clinic. *Sex Transm Dis*. 2018;45(10):696-702. doi:10.1097/OLQ.0000000000000854

13. Bhat V, Tazari M, Watt KD, Bhat M. New-Onset Diabetes and Preexisting Diabetes Are Associated With Comparable Reduction in Long-Term Survival After Liver Transplant: A Machine Learning Approach. *Mayo Clin Proc*. 2018;93(12):1794-1802. doi:10.1016/j.mayocp.2018.06.020

14. Choi HW, Park HJ, Choi SY, et al. Early prediction of the severity of acute pancreatitis using radiologic and clinical scoring systems with classification tree analysis. *Am J Roentgenol*. 2018;211(5):1035-1043. doi:10.2214/AJR.18.19545

15. Ogunleye A, Wang QG. XGBoost Model for Chronic Kidney Disease Diagnosis. *IEEE/ACM Trans Comput Biol Bioinforma*. 2020;17(6):2131-2140. doi:10.1109/TCBB.2019.2911071

16. Wu CC, Yeh WC, Hsu WD, et al. Prediction of fatty liver disease using machine learning algorithms. *Comput Methods Programs Biomed*. 2019;170:23-29. doi:10.1016/j.cmpb.2018.12.032

17. Shigemi D, Yamaguchi S, Aso S, Yasunaga H. Predictive model for macrosomia using maternal parameters without sonography information. *J Matern Neonatal Med*. 2019;32(22):3859-3863. doi:10.1080/14767058.2018.1484090

18. Friedman KG, Sleeper LA, Freud LR, et al. Improved technical success, postnatal outcome and refined predictors of outcome for fetal aortic valvuloplasty. *Ultrasound Obstet Gynecol*. 2018;52(2):212-220. doi:10.1002/uog.17530

19. Duan H, Sun Z, Dong W, Huang Z. Utilizing dynamic treatment information for MACE prediction of acute coronary syndrome. *BMC Med Inform Decis Mak*. 2019;19(1):1-11. doi:10.1186/s12911-018-0730-7

20. Ansart M, Epelbaum S, Gagliardi G, et al. Reduction of recruitment costs in preclinical AD trials: validation of automatic pre-screening algorithm for brain amyloidosis. *Stat Methods Med Res*. 2020;29(1):151-164. doi:10.1177/0962280218823036

21. Kwon J myoung, Jeon KH, Kim HM, et al. Deep-learning-based out-of-hospital cardiac arrest prognostic system to predict clinical outcomes. *Resuscitation*. 2019;139(April 2019):84-91. doi:10.1016/j.resuscitation.2019.04.007

22. Hammond R, Athanasiadou R, Curado S, et al. Correction: Predicting childhood obesity using electronic health records and publicly available data. *PLoS One*. 2019;14(10):1-18. doi:10.1371/journal.pone.0223796

23. Zamboni S, Moschini M, Antonelli A, et al. How to improve patient selection for neoadjuvant chemotherapy in bladder cancer patients candidate for radical cystectomy and pelvic lymph node dissection. *World J Urol*. Published online August 2019. doi:10.1007/s00345-019-02916-2

24. Nobles AL, Glenn JJ, Barnes LE. Identification of Inminent Suicide Risk Among Young Adults using Text Messages. *Proc SIGCHI Conf Hum Factor Comput Syst*. Published online 2019:1-22. doi:10.1145/3173574.3173987.Identification

25. Tse R, Garland J, Kesha K, et al. Combining Postmortem Vitreous Sodium and Chloride and Lung-Body Ratio in Aiding the Diagnosing Saltwater Drowning. *Am J Forensic Med Pathol*. 2018;39(3):229-235. doi:10.1097/PAF.0000000000000410

26. Sterling NW, Patzer RE, Mengyu D, et. a.Prediction of emergency department patient disposition based on natural language processing of triage notes. *Int J Med Inform*. 2019;129:184-188. doi:10.1016/j.ijmedinf.2019.06.008

27. Perveen S, Shahbaz M, Keshavjee K, Guergachi A. A Systematic Machine Learning Based Approach for the Diagnosis of Non-Alcoholic Fatty Liver Disease Risk and Progression. *Sci Rep*. 2018;8(1):1-12. doi:10.1038/s41598-018-20166-x

28. Pei Z, Liu J, Liu M, et al. Risk-Predicting Model for Incident of

Essential Hypertension Based on Environmental and Genetic Factors with Support Vector Machine. *Interdiscip Sci Comput Life Sci*. 2018;10(1):126-130. doi:10.1007/s12539-017-0271-2

29. Bouallègue F Ben, Mariano-Goulart D, Payoux P. Joint Assessment of Quantitative 18 F-Florbetapir and 18 F-FDG Regional Uptake Using Baseline Data from the ADNI. *J Alzheimer's Dis*. 2018;62(1):399-408. doi:10.3233/JAD-170833

30. Tsai TL, Huang MH, Lee CY, et. al. Data Science for Extubation Prediction and Value of Information in Surgical Intensive Care Unit. *J Clin Med*. 2019;8(10). doi:10.3390/jcm8101709

31. Lopes RR, van Mourik MS, Schaft E V., et al. Value of machine learning in predicting TAVI outcomes. *Netherlands Hear J*. 2019;27(9):443-450. doi:10.1007/s12471-019-1285-7

32. Maragatham G, Devi S. LSTM Model for Prediction of Heart Failure in Big Data. *J Med Syst*. 2019;43(5). doi:10.1007/s10916-019-1243-3

33. Shao CY, Liu KC, Li CL, et al. C-reactive protein to albumin ratio is a key indicator in a predictive model for anastomosis leakage after esophagectomy: Application of classification and regression tree analysis. *Thorac Cancer*. 2019;10(4):728-737. doi:10.1111/1759-7714.12990

34. Saccà V, Sarica A, Novellino F, et al. Evaluation of machine learning algorithms performance for the prediction of early multiple sclerosis from resting-state FMRI connectivity data. *Brain Imaging Behav*. 2019;13(4):1103-1114. doi:10.1007/s11682-018-9926-9

35. Cearns M, Opel N, Clark S, et al. Predicting rehospitalization within 2 years of initial patient admission for a major depressive episode: a multimodal machine learning approach. *Transl Psychiatry*. 2019;9(1). doi:10.1038/s41398-019-0615-2

36. Bouts MJRJ, van der Grond J, Vernooij MW, et al. Detection of mild cognitive impairment in a community-dwelling population using quantitative, multiparametric MRI-based classification. *Hum Brain Mapp*. 2019;40(9):2711-2722. doi:10.1002/hbm.24554

37. Huben NB, Hussein AA, May PR, et al. Development of a Patient-Based Model for Estimating Operative Times for Robot-Assisted Radical Prostatectomy. *J Endourol*. 2018;32(8):730-736. doi:10.1089/end.2018.0249

38. Hale AT, Stonko DP, Brown A, et al. Machine-learning analysis outperforms conventional statistical models and CT classification systems in predicting 6-month outcomes in pediatric patients sustaining traumatic brain injury. *Neurosurg Focus*. 2018;45(5):1-7. doi:10.3171/2018.8.FOCUS17773

39. Salvatore C, Castiglioni I. A wrapped multi-label classifier for the automatic diagnosis and prognosis of Alzheimer's disease. *J Neurosci Methods*. 2018;302:58-65. doi:10.1016/j.jneumeth.2017.12.016

40. Yang H, Bath PA. The Use of Data Mining Methods for the Prediction of Dementia: Evidence from the English Longitudinal Study of Aging. *IEEE J Biomed Heal Informatics*. 2020;24(2):345-353. doi:10.1109/JBHI.2019.2921418

41. Zhou M, Fukuoka Y, Goldberg K, Vittinghoff E, Aswani A. Applying machine learning to predict future adherence to physical activity programs. *BMC Med Inform Decis Mak*. 2019;19(1):0-11. doi:10.1186/s12911-019-0890-0

42. Kang X, Liang Y, Wang S, et al. Prediction model comparison for gestational diabetes mellitus with macrosomia based on risk factor investigation. *J Matern Neonatal Med*. 2019;0(0):1-10. doi:10.1080/14767058.2019.1668922

43. Sauer CM, Sasson D, Paik KE, et al. Feature selection and prediction of treatment failure in tuberculosis. *PLoS One*. 2018;13(11):1-14. doi:10.1371/journal.pone.0207491

44. Thornblade LW, Flum DR, Flaxman AD. Predicting Future Elective Colon Resection for Diverticulitis Using Patterns of Health Care Utilization. *eGEMs (Generating Evid Methods to Improv patient outcomes)*. 2018;6(1):1. doi:10.5334/egems.193

45. Lei VJ, Kennedy EH, Luong T, et al. Model performance metrics in assessing the value of adding intraoperative data for death prediction: Applications to noncardiac surgery. *Stud Health Technol Inform*. 2019;264:223-227. doi:10.3233/SHTI190216

46. Lee SJ, Yu SH, Kim Y, et al. Development of integrated data and prediction system platform for the localized prostate cancer. *Stud Health Technol Inform*. 2019;264:1506-1507. doi:10.3233/SHTI190507

47. Auffenberg GB, Ghani KR, Ramani S, et al. askMUSIC: Leveraging a Clinical Registry to Develop a New Machine Learning Model to Inform Patients of Prostate Cancer Treatments Chosen by Similar Men. *Eur Urol*. 2019;75(6):901-907. doi:10.1016/j.eururo.2018.09.050

48. Wang Z, Wang B, Zhou Y, Li D, Yin Y. Weight-based multiple empirical kernel learning with neighbor discriminant constraint for heart failure mortality prediction. *J Biomed Inform*. 2020;101(November 2018):103340. doi:10.1016/j.jbi.2019.103340

49. Nelson A, Herron D, Rees G, Nachev P. Predicting scheduled hospital attendance with artificial intelligence. *npj Digit Med*. 2019;2(1):1-7. doi:10.1038/s41746-019-0103-3

50. Shibahara T, Ikuta S, Muragaki Y. Machine-Learning Approach for Modeling Myelosuppression Attributed to Nimustine Hydrochloride. *JCO Clin Cancer Informatics*. 2018;(2):1-21. doi:10.1200/cci.17.00022

51. Liang S, Brown MRG, Deng W, et al. Convergence and divergence of neurocognitive patterns in schizophrenia and depression. *Schizophr Res*. 2018;192:327-334. doi:10.1016/j.schres.2017.06.004

52. Chen R, Stewart WF, Sun J, Ng K, Yan X. Recurrent neural networks for early detection of heart failure from longitudinal electronic health record data: Implications for temporal modeling with respect to time before diagnosis, data density, data quantity, and data type. *Circ Cardiovasc Qual Outcomes*. 2019;12(10):1-15. doi:10.1161/CIRCOUTCOMES.118.005114

53. Klang E, Barash Y, Soffer S, et al. Promoting head CT exams in the emergency department triage using a machine learning model. *Neuroradiology*. 2020;62(2):153-160. doi:10.1007/s00234-019-02293-y

54. Fan Y, Li Y, Li Y, et al. Development and assessment of machine learning algorithms for predicting remission after transsphenoidal surgery among patients with acromegaly. *Endocrine*. 2020;67(2):412-422. doi:10.1007/s12020-019-02121-6

55. Ferré Á, Poca MA, De La Calzada MD, et al. A conditional inference tree model for predicting sleep-related breathing disorders in patients with Chiari malformation type 1: Description and external validation. *J Clin Sleep Med*. 2019;15(1):89-99. doi:10.5664/jcsm.7578

56. Zhang F, Zhang Y, Ke C, et al. Predicting ovarian cancer recurrence by plasma metabolic profiles before and after surgery. *Metabolomics*. 2018;14(5):1-9. doi:10.1007/s11306-018-1354-8

57. Ferrer-Peña R, Calvo-Lobo C, Gómez M, Muñoz-García D. Prediction Model for Choosing Needle Length to Minimize Risk of Median Nerve Puncture With Dry Needling of the Pronator Teres. *J Manipulative Physiol Ther*. 2019;42(5):366-371. doi:10.1016/j.jmpt.2018.11.020

58. Cameron JM, Butler HJ, Smith BR, et al. Developing infrared spectroscopic detection for stratifying brain tumour patients: Glioblastoma multiforme: Vs. lymphoma. *Analyst*. 2019;144(22):6736-6750. doi:10.1039/c9an01731c

59. Harris AH, Kuo AC, Bowe T, Gupta S, Nordin D, Giori NJ. Prediction Models for 30-Day Mortality and Complications After Total Knee and Hip Arthroplasties for Veteran Health Administration Patients With Osteoarthritis. *J Arthroplasty*. 2018;33(5):1539-1545. doi:10.1016/j.arth.2017.12.003

60. Kuo KM, Talley PC, Huang CH, Cheng LC. Predicting hospital-acquired pneumonia among schizophrenic patients: A machine learning approach. *BMC Med Inform Decis Mak*. 2019;19(1):1-8. doi:10.1186/s12911-019-0792-1

61. Arai Y, Kondo T, Fuse K, et al. Using a machine learning algorithm to predict acute graft-versus-host disease following allogeneic transplantation. *Blood Adv*. 2019;3(22):3626-3634. doi:10.1182/bloodadvances.2019000934

62. Paul JK, Iype T, R D, Hagiwara Y, Koh JEW, Acharya UR. Characterization of fibromyalgia using sleep EEG signals with nonlinear dynamical features. *Comput Biol Med*. 2019;111(April):103331. doi:10.1016/j.compbiomed.2019.103331

63. Liu L, Yu Y, Fei Z, et al. An interpretable boosting model to predict side effects of analgesics for osteoarthritis. *BMC Syst Biol*. 2018;12(Suppl 6). doi:10.1186/s12918-018-0624-4

64. Shappell C, Snyder A, Edelson DP, et. al. Predictors of In-hospital Mortality after rapid response team calls in a 274 hospital nationwide sample. *Crit Care Med*. 2018;176(10):139-148. doi:10.1097/CMM.0000000000002926

65. Zhang R, Jing Y, Zhang H, et al. Comprehensive Evolutionary Analysis of the Major RNA-Induced Silencing Complex Members. *Sci Rep*. 2018;8(1):14189. doi:10.1038/s41598-018-32635-4

66. Ge F, Li Y, Yuan M, Zhang J, Zhang W. Identifying predictors of probable posttraumatic stress disorder in children and adolescents with earthquake exposure: A longitudinal study using a machine learning approach. *J Affect Disord*. 2020;264(September 2019):483-493. doi:10.1016/j.jad.2019.11.079

67. Dhondt B, De Bleser E, Claeys T, et al. Discovery and validation of a serum microRNA signature to characterize oligo- and polymetastatic prostate cancer: not ready for prime time. *World J Urol*. 2019;37(12):2557-2564. doi:10.1007/s00345-018-2609-8

68. Rohaut B, Doyle KW, Reynolds AS, et al. Deep structural brain lesions associated with consciousness impairment early after hemorrhagic stroke. *Sci Rep*. 2019;9(1):1-9. doi:10.1038/s41598-019-41042-2

69. Karnuta JM, Navarro SM, Haeberle HS, et al. Predicting Inpatient Payments Prior to Lower Extremity Arthroplasty Using Deep Learning: Which Model Architecture Is Best? *J Arthroplasty*. 2019;34(10):2235-2241.e1. doi:10.1016/j.arth.2019.05.048

70. Wilson MB, Ali SA, Kovatch KJ, Smith JD, Hoff PT. Machine Learning Diagnosis of Peritonsillar Abscess. *Otolaryngol - Head Neck Surg (United States)*. 2019;161(5):796-799.

doi:10.1177/0194599819868178

71. Lu B, Fu L, Nie B, Peng Z, Liu H. A novel framework with high diagnostic sensitivity for lung cancer detection by electronic nose. *Sensors (Switzerland)*. 2019;19(23):1-29. doi:10.3390/s19235333

72. Xu Y, Kong S, Cheung WY, et al. Development and validation of case-finding algorithms for recurrence of breast cancer using routinely collected administrative data. *BMC Cancer*. 2019;19(1):1-10. doi:10.1186/s12885-019-5432-8

73. Cohen S, Fulcher BD, Rajaratnam SMW, et al. Sleep patterns predictive of daytime challenging behavior in individuals with low-functioning autism. *Autism Res*. 2018;11(2):391-403. doi:10.1002/aur.1899

74. Wang Y, Liu W, Yu Y, et al. Prediction of the Depth of Tumor Invasion in Gastric Cancer: Potential Role of CT Radiomics. *Acad Radiol*. 2019;(6):1-8. doi:10.1016/j.acra.2019.10.020

75. Mortezagholi A, Khosravizadehorcid O, Menhaj MB, Shafigh Y, Kalhor R. Make intelligent of gastric cancer diagnosis error in Qazvin's medical centers: Using data mining method. *Asian Pacific J Cancer Prev*. 2019;20(9):2607-2610. doi:10.31557/APJCP.2019.20.9.2607

76. Muehlematter UJ, Mannil M, Becker AS, et al. Vertebral body insufficiency fractures: detection of vertebrae at risk on standard CT images using texture analysis and machine learning. *Eur Radiol*. 2019;29(5):2207-2217. doi:10.1007/s00330-018-5846-8

77. Faruqui SHA, Du Y, Meka R, et al. Development of a deep learning model for dynamic forecasting of blood glucose level for type 2 diabetes mellitus: Secondary analysis of a randomized controlled trial. *JMIR mHealth uHealth*. 2019;7(11):1-14. doi:10.2196/14452

78. Molinari M, Ayloo S, Tsung A, et al. Prediction of Perioperative Mortality of Cadaveric Liver Transplant Recipients during Their Evaluations. *Transplantation*. 2019;103(10):E297-E307. doi:10.1097/TP.0000000000002810

79. Jeon JP, Kim C, Oh BD, Kim SJ, Kim YS. Prediction of persistent hemodynamic depression after carotid angioplasty and stenting using artificial neural network model. *Clin Neurol Neurosurg*. 2018; 127-131. doi:10.1016/j.clineuro.2017.12.005

80. Staartjes VE, Serra C, Muscas G, et al. Utility of deep neural networks in predicting gross-total resection after transsphenoidal surgery for pituitary adenoma: A pilot study. *Neurosurg Focus*. 2018;45(5):1-7. doi:10.3171/2018.8.FOCUS18243

81. Lu CF, Hsu FT, Hsieh KLC, et al. Machine learning–based radiomics for molecular subtyping of gliomas. *Clin Cancer Res*. 2018;24(18):4429-4436. doi:10.1158/1078-0432.CCR-17-3445

82. Park N, Kang E, Park M, et al. Predicting acute kidney injury in cancer patients using heterogeneous and irregular data. *PLoS One*. 2018;13(7):1-21. doi:10.1371/journal.pone.0199839

83. Ballarini T, Mueller K, Albrecht F, et al. Regional gray matter changes and age predict individual treatment response in Parkinson's disease. *NeuroImage Clin*. 2019;21(May 2018). doi:10.1016/j.nicl.2018.101636

84. Chen D, Goyal G, Go RS, Parikh SA, Ngufor CG. Improved Interpretability of Machine Learning Model Using Unsupervised Clustering: Predicting Time to First Treatment in Chronic Lymphocytic Leukemia. *JCO Clin Cancer Informatics*. 2019;(3):1-11. doi:10.1200/cci.18.00137

85. Malycha J, Farajidavar N, Pimentel MAF, et al. The effect of fractional inspired oxygen concentration on early warning score performance: A database analysis. *Resuscitation*. 2019;139(April 2019):192-199. doi:10.1016/j.resuscitation.2019.04.002

86. Xie Z, Nikolayeva O, Luo J, Li D. Building risk prediction models for type 2 diabetes using machine learning techniques. *Prev Chronic Dis*. 2019;16(9):1-9. doi:10.5888/pcd16.190109

87. Thanathornwong B. Bayesian-based decision support system for assessing the needs for orthodontic treatment. *Healthc Inform Res*. 2018;24(1):22-28. doi:10.4258/hir.2018.24.1.22

88. Ngo CQ, Truong BCQ, Jones TW, Nguyen HT. Occipital EEG Activity for the Detection of Nocturnal Hypoglycemia. *Proc Annu Int Conf IEEE Eng Med Biol Soc EMBS*. 2018;2018-July:3862-3865. doi:10.1109/EMBC.2018.8513069

89. Hyun SH, Ahn MS, Koh YW, et.al. A Machine-Learning Approach Using PET-Based Radiomics to Predict the Histological Subtypes of Lung Cancer. *Clin Nucl Med*. 2019;44(12):956-960. doi:10.1097/RLU.0000000000002810

90. Rozet A, Kronish IM, Schwartz JE, Davidson KW. Using machine learning to derive just-in-time and personalized predictors of stress: Observational study bridging the gap between nomothetic and ideographic approaches. *J Med Internet Res*. 2019;21(4):1-16. doi:10.2196/12910

91. Mellem MS, Liu Y, Gonzalez H, Kollada M, Martin WJ, Ahammad P. Machine Learning Models Identify Multimodal Measurements Highly Predictive of Transdiagnostic Symptom Severity for Mood, Anhedonia, and Anxiety. *Biol Psychiatry Cogn Neurosci Neuroimaging*. 2020;5(1):56-67. doi:10.1016/j.bpsc.2019.07.007

92. Karhade A V., Ogink PT, Thio QCBS, et al. Development of machine learning algorithms for prediction of prolonged opioid prescription after surgery for lumbar disc herniation. *Spine J*. 2019;19(11):1764-1771. doi:10.1016/j.spinee.2019.06.002

93. Zhang F, Tian S, Chen S, Ma Y, Li X, Guo X. Voxel-Based Morphometry: Improving the Diagnosis of Alzheimer's Disease Based on an Extreme Learning Machine Method from the ADNI cohort. *Neuroscience*. 2019;414:273-279. doi:10.1016/j.neuroscience.2019.05.014

94. Leightley D, Williamson V, Darby J, Fear NT. Identifying probable post-traumatic stress disorder: applying supervised machine learning to data from a UK military cohort. *J Ment Heal*. 2019;28(1):34-41. doi:10.1080/09638237.2018.1521946

95. Mulder M, Nijland RH, van de Port IG, van Wegen EE, Kwakkel G. Prospectively Classifying Community Walkers After Stroke: Who Are They? *Arch Phys Med Rehabil*. 2019;100(11):2113-2118. doi:10.1016/j.apmr.2019.04.017

96. Debédat J, Sokolovska N, Coupaye M, et al. Long-term Relapse of Type 2 Diabetes After Roux-en-Y Gastric Bypass: Prediction and clinical relevance. *Diabetes Care*. 2018;41(10):2086-2095. doi:10.2337/dc18-0567

97. Alcantud JCR, Varela G, Santos-Buitrago B, Santos-García G, Jiménez MF. Analysis of survival for lung cancer resections cases with fuzzy and soft set theory in surgical decision making. *PLoS One*. 2019;14(6):1-17. doi:10.1371/journal.pone.0218283

98. Sandström A, Snowden JM, Höijer J, Bottai M, Wikström AK. Clinical risk assessment in early pregnancy for preeclampsia in nulliparous women: A population based cohort study. *PLoS One*. 2019;14(11):1-16. doi:10.1371/journal.pone.0225716

99. Xiao C, Liu Y, Feng DD, Wang X. Key Marker Selection for the Detection of Early Parkinson' s Disease using Importance-Driven Models. *Conf Proc . Annu Int Conf IEEE Eng Med Biol Soc IEEE Eng Med Biol Soc Annu Conf*. 2018;2018:6100-6103. doi:10.1109/EMBC.2018.8513564

100. Cooper JN, Minneci PC, Deans KJ. Postoperative neonatal mortality prediction using superlearning. *J Surg Res*. 2018;221:311-319. doi:10.1016/j.jss.2017.09.002

101. Rau CS, Kuo PJ, Chien PC, Huang CY, Hsieh HY, Hsieh CH. Mortality prediction in patients with isolated moderate and severe traumatic brain injury using machine learning models. *PLoS One*. 2018;13(11):1-12. doi:10.1371/journal.pone.0207192

102. Anand RS, Stey P, Jain S, et al. Predicting Mortality in Diabetic ICU Patients Using Machine Learning and Severity Indices. *AMIA Jt Summits Transl Sci proceedings AMIA Jt Summits Transl Sci*. 2018;2017:310-319.

103. Aperstein Y, Cohen L, Bendavid I, et al. Improved ICU mortality prediction based on SOFA scores and gastrointestinal parameters. *PLoS One*. 2019;14(9):1-11. doi:10.1371/journal.pone.0222599

104. Park J, Kim JW, Ryu B, Heo E, Jung SY, Yoo S. Patient-level prediction of cardio-cerebrovascular events in hypertension using nationwide claims data. *J Med Internet Res*. 2019;21(2). doi:10.2196/11757

105. Gökçay D, Eken A, Baltaci S. Binary Classification Using Neural and Clinical Features: An Application in Fibromyalgia with Likelihood-Based Decision Level Fusion. *IEEE J Biomed Heal Informatics*. 2019;23(4):1490-1498. doi:10.1109/JBHI.2018.2844300

106. Gupta R, Gupta S, Chauhan L. Predicting visual outcome after open globe injury using classification and regression tree model: the Moradabad ocular trauma study. *Can J Ophthalmol*. 2019;54(4):473-478. doi:10.1016/j.jcjo.2018.08.004

107. Butt AH, Rovini E, Dolciotti C, et al. Objective and automatic classifcation of Parkinson disease with Leap Motion controller. *Biomed Eng Online*. 2018;17(1):1-21. doi:10.1186/s12938-018-0600-7

108. Hunter-Zinck HS, Peck JS, Strout TD, Gaehde SA. Predicting emergency department orders with multilabel machine learning techniques and simulating effects on length of stay. *J Am Med Informatics Assoc*. 2019;26(12):1427-1436. doi:10.1093/jamia/ocz171

109. Kilic A, Goyal A, Miller JK, et al. Predictive Utility of a Machine Learning Algorithm in Estimating Mortality Risk in Cardiac Surgery. *Ann Thorac Surg*. Published online 2020. doi:10.1016/j.athoracsur.2019.09.049

110. Campillo-Artero C, Serra-Burriel M, Calvo-Pérez A. Predictive modeling of emergency cesarean delivery. *PLoS One*. 2018;13(1):1-14. doi:10.1371/journal.pone.0191248

111. Zhang ML, Guo AX, Kadauke S, Dighe AS, Baron JM, Sohani AR. Machine Learning Models Improve the Diagnostic Yield of Peripheral Blood Flow Cytometry. *Am J Clin Pathol*. 2020;153(2):235-242. doi:10.1093/ajcp/aqz150

112. Hong WS, Haimovich AD, Taylor RA. Predicting hospital admission at emergency department triage using machine learning. *PLoS One*. 2018;13(7):1-13. doi:10.1371/journal.pone.0201016

113. Beauchet O, Noublanche F, Simon R, et. al. Falls risk prediction

for older inpatients in acute care medical wards: is there an interest to combine an early nurse assessment and the artificial neural network analysis? *J Nutr Health Aging*. 2018. doi:10.1007/s12603-017-0950-z

114. Ma Z, Wang P, Gao Z, Wang R, Khalighi K. Ensemble of machine learning algorithms using the stacked generalization approach to estimate the warfarin dose. *PLoS One*. 2018;13(10):1-12. doi:10.1371/journal.pone.0205872

115. Veeranki SPK, Hayn D, Jauk S, et al. An improvised classification model for predicting delirium. *Stud Health Technol Inform*. 2019;264:1566-1567. doi:10.3233/SHTI190537

116. Maharlou H, Niakan Kalhori SR, Shahbazi S, Ravangard R. Predicting length of stay in intensive care units after cardiac surgery: Comparison of artificial neural networks and adaptive neuro-fuzzy system. *Healthc Inform Res*. 2018;24(2):109-117. doi:10.4258/hir.2018.24.2.109

117. Castillo-Olea C, B G-ZS, C CL, Zuniga C. Automatic Classification of Sarcopenia Level in Older Adults: A Case Study at Tijuana General Hospital. *Int J Environ Res Public Health*. 2019;16(18). doi:10.3390/ijerph16183275

118. Sa-ngamuang C, Haddawy P, Luvira V, et al. Accuracy of dengue clinical diagnosis with and without NS1 antigen rapid test: Comparison between human and Bayesian network model decision. *PLoS Negl Trop Dis*. 2018;12(6):1-14. doi:10.1371/journal.pntd.0006573

119. Talaei-Khoei A, Wilson JM. Identifying people at risk of developing type 2 diabetes: A comparison of predictive analytics techniques and predictor variables. *Int J Med Inform*. 2018;119(January):22-38. doi:10.1016/j.ijmedinf.2018.08.008

120. Bertsimas D, Dunn J, Velmahos GC, Kaafarani HMA. Surgical Risk Is Not Linear: Derivation and Validation of a Novel, User-friendly, and Machine-learning-based Predictive OpTimal Trees in Emergency Surgery Risk (POTTER) Calculator. *Ann Surg*. 2018;268(4):574-583. doi:10.1097/SLA.0000000000002956

121. Liu C, Qi L, Feng QX, Sun SW, Zhang YD, Liu XS. Performance of a machine learning-based decision model to help clinicians decide the extent of lymphadenectomy (D1 vs. D2) in gastric cancer before surgical resection. *Abdom Radiol*. 2019;44(9):3019-3029. doi:10.1007/s00261-019-02098-w

122. Luo G, Stone BL, Nkoy FL, He S, Johnson MD. Predicting appropriate hospital admission of emergency department patients with bronchiolitis: Secondary analysis. *JMIR Med Informatics*. 2019;7(1):1-15. doi:10.2196/12591

123. Meena K, Tayal DK, Gupta V, Fatima A. Using classification techniques for statistical analysis of Anemia. *Artif Intell Med*. 2019;94(August 2018):138-152. doi:10.1016/j.artmed.2019.02.005

124. Takeuchi M, Kawakubo H, Mayanagi S, et al. Postoperative Pneumonia is Associated with Long-Term Oncologic Outcomes of Definitive Chemoradiotherapy Followed by Salvage Esophagectomy for Esophageal Cancer. *J Gastrointest Surg*. 2018;22(11):1881-1889. doi:10.1007/s11605-018-3857-z

125. Kiiski H, Jollans L, SO D, et al. Machine Learning EEG to Predict Cognitive Functioning and Processing Speed Over a 2-Year Period in Multiple Sclerosis Patients and Controls. *Brain Topogr*. 2018;31(3):346-363. doi:10.1007/s10548-018-0620-4

126. Hasnain Z, Mason J, Gill K, et al. Machine learning models for predicting post-cystectomy recurrence and survival in bladder cancer patients. *PLoS One*. 2019;14(2):1-15. doi:10.1371/journal.pone.0210976

127. Dean J, Wong K, Gay H, et al. Incorporating spatial dose metrics in machine learning-based normal tissue complication probability (NTCP) models of severe acute dysphagia resulting from head and neck radiotherapy. *Clin Transl Radiat Oncol*. 2018;8:27-39. doi:10.1016/j.ctro.2017.11.009

128. Cheung M, Cobb AN, Kuo PC. Predicting burn patient mortality with electronic medical records. *Surg (United States)*. 2018;164(4):839-847. doi:10.1016/j.surg.2018.07.010

129. Gowin JL, Ernst M, Ball T, et al. Using neuroimaging to predict relapse in stimulant dependence: A comparison of linear and machine learning models. *NeuroImage Clin*. 2019;21(January):101676. doi:10.1016/j.nicl.2019.101676

130. Rojas JC, Carey KA, Edelson DP, Venable LR, Howell MD, Churpek MM. Predicting intensive care unit readmission with machine learning using electronic health record data. *Ann Am Thorac Soc*. 2018;15(7):846-853. doi:10.1513/AnnalsATS.201710-787OC

131. Wei R, Lin K, Yan W, et al. Computer-Aided Diagnosis of Pancreas Serous Cystic Neoplasms: A Radiomics Method on Preoperative MDCT Images. *Technol Cancer Res Treat*. 2019;18:1533033818824339. doi:10.1177/1533033818824339

132. Balani J, Hyer SL, Shehata H, Mohareb F. Visceral fat mass as a novel risk factor for predicting gestational diabetes in obese pregnant women. *Obstet Med*. 2018;11(3):121-125. doi:10.1177/1753495X17754149

133. Gao L, Smielewski P, Li P, Czosnyka M, Ercole A. Signal Information Prediction of Mortality Identifies Unique Patient Subsets after Severe Traumatic Brain Injury: A Decision-Tree Analysis Approach. *J Neurotrauma*. doi:10.1089/neu.2019.6631

134. Garcia-Arce A, Rico F, Zayas-Castro JL. Comparison of Machine Learning Algorithms for the Prediction of Preventable Hospital Readmissions. *J Healthc Qual*. 2018;40(3):129-138. doi:10.1097/JHQ.0000000000000080

135. Papini S, Pisner D, Shumake J, et al. Ensemble machine learning prediction of posttraumatic stress disorder screening status after emergency room hospitalization. *J Anxiety Disord*. 2018;60(October):35-42. doi:10.1016/j.janxdis.2018.10.004

136. Jang BS, Jeon SH, Kim IH, Kim IA. Prediction of Pseudoprogression versus Progression using Machine Learning Algorithm in Glioblastoma. *Sci Rep*. 2018;8(1):1-9. doi:10.1038/s41598-018-31007-2

137. Cosgriff C V., Celi LA, Ko S, et al. Developing well-calibrated illness severity scores for decision support in the critically ill. *npj Digit Med*. 2019;2(1). doi:10.1038/s41746-019-0153-6

138. Kim H, Lee SH, Lee SE, Hong S, Kang HJ, Kim N. Depression prediction by using ecological momentary assessment, actiwatch data, and machine learning: Observational study on older adults living alone. *JMIR mHealth uHealth*. 2019;7(10). doi:10.2196/14149

139. Lötsch J, Sipilä R, Tasmuth T, et al. Machine-learning-derived classifier predicts absence of persistent pain after breast cancer surgery with high accuracy. *Breast Cancer Res Treat*. 2018;171(2):399-411. doi:10.1007/s10549-018-4841-8

140. Spyroglou II, Spöck G, Rigas AG, Paraskakis EN. Evaluation of Bayesian classifiers in asthma exacerbation prediction after medication discontinuation. *BMC Res Notes*. 2018;11(1):18-23. doi:10.1186/s13104-018-3621-1

141. Goudey B, Fung BJ, Schieber C, et al. A blood-based signature of cerebrospinal fluid Aβ 1–42 status. *Sci Rep*. 2019;9(1):0-12.

doi:10.1038/s41598-018-37149-7

142. Facciorusso A, Del Prete V, Antonino M, Buccino VR, Muscatiello N. Response to repeat echoendoscopic celiac plexus neurolysis in pancreatic cancer patients: A machine learning approach. *Pancreatology*. 2019;19(6):866-872. doi:10.1016/j.pan.2019.07.038

143. Kim DW, Kim H, Nam W, Kim HJ, Cha IH. Machine learning to predict the occurrence of bisphosphonate-related osteonecrosis of the jaw associated with dental extraction: A preliminary report. *Bone*. 2018;116(January):207-214. doi:10.1016/j.bone.2018.04.020

144. Karhade A V., Shah AA, Bono CM, et al. Development of machine learning algorithms for prediction of mortality in spinal epidural abscess. *Spine J*. 2019;19(12):1950-1959. doi:10.1016/j.spinee.2019.06.024

145. Tu W, Chen PA, Koenig N, et al. Machine learning models reveal neurocognitive impairment type and prevalence are associated with distinct variables in HIV/AIDS. *J Neurovirol*. 2020;26(1):41-51. doi:10.1007/s13365-019-00791-6

146. Van Steenkiste T, Ruyssinck J, De Baets L, et al. Accurate prediction of blood culture outcome in the intensive care unit using long short-term memory neural networks. *Artif Intell Med*. 2019;97(April 2017):38-43. doi:10.1016/j.artmed.2018.10.008

147. Paliwal N, Jaiswal P, Tutino VM, et al. Outcome prediction of intracranial aneurysm treatment by flow diverters using machine learning. *Neurosurg Focus*. 2018;45(5):1-10. doi:10.3171/2018.8.FOCUS18332

148. Bronsert M, Singh AB, Henderson WG, Hammermeister K, Meguid RA, Colborn KL. Identification of postoperative complications using electronic health record data and machine learning. *Am J Surg*. 2020;220(1):114-119. doi:10.1016/j.amjsurg.2019.10.009

149. Eill A, Jahedi A, Gao Y, et al. Functional Connectivities Are More Informative Than Anatomical Variables in Diagnostic Classification of Autism. *Brain Connect*. 2019;9(8):604-612. doi:10.1089/brain.2019.0689

150. Cook F, Lobo D, Martin M, et al. Prospective validation of a new airway management algorithm and predictive features of intubation difficulty. *Br J Anaesth*. 2019;122(2):245-254. doi:10.1016/j.bja.2018.09.021

151. Eggleston B, Dismuke-Greer CE, Pogoda TK, et al. A prediction model of military combat and training exposures on VA service-connected disability: a CENC study. *Brain Inj*. 2019;33(13-14):1602-1614. doi:10.1080/02699052.2019.1655793

152. Villarreal YR, Suchting R, Klawans MR, et al. Predicting HCV Incidence in Latinos with High-Risk Substance Use: A Data Science Approach. *Soc Work Public Health*. 2019;34(7):606-615. doi:10.1080/19371918.2019.1635948

List of assessed supervised machine learning techniques

Adaptive Boosting (Adaboost)
ADTree
Bagged Classification Tree
Bayesian Network
CatBoost
Boosted regression
Classification and regression tree (CART)
Conditional inference tree (URP-CTREE)
Dense Convolutional Network (DenseNet)
Elastic Net
Gaussian Naïve Bayes (GMB)
Generalized Linear Model (GLM)
Gradient Boosting Decision Tree (GBDT)
Gradient Boosting Machine (GBM)
Keras-based convolutional neural network (K-CNN)
K-Nearest Neighbour
LASSO
Light gradient boosting machine (LGB)
Linear regression
Logistic Regression
Logitboost
Long short-term memory (LSTM)
Multilayer perceptron (MLP)
Multiple Kernel Learning (MultiK-MHKS)
Nadir-weighted SVM (NwSVM)
Naïve Bayes (NB)
Neural Network (NN)
Optimal Classification Tree (OCT)
Random Forest (RF)
Random Survival Forest (RSF)
Regularized Greedy forests (RGF)
Ridge Regression
RUSBoost
Support Vector Machine (SVM)
Tree-augmented Naive Bayes

Supplemental file 4: Characteristics of included studies stratified by study type

Table S2. PROBAST Signaling questions for model development and validation analyses in 94 included prognostic studies

		Developed models (n=94)			External validations (n=12)		
		Yes, probably yes *n* (%; 95% CI)	No, probably no *n* (%; 95% CI)	No information *n* (%; 95% CI)	Yes, probably yes *n* (%; 95% CI)	No, probably no *n* (%; 95% CI)	No information *n* (%; 95% CI)
Participants							
1.1	Were appropriate data sources used, e.g. cohort, RCT or nested case-control study data?	70 (75, 65 to 82)	21 (22, 15 to 32)	3 (3, 1 to 9)	10 (83, 55 to 95)	2 (17, 5 to 45)	0 (0.0)
1.2	Were all inclusions and exclusions of participants appropriate?	57 (61, 51 to 70)	12 (13, 8 to 21)	25 (27, 19 to 36)	5 (41, 19 to 68)	0 (0.0)	7 (58, 32 to 81)
Predictors							
2.1	Were predictors defined and assessed in a similar way for all participants?	63 (67, 57 to 76)	14 (15, 9 to 24)	17 (18, 11 to 27)	3 (25, 9 to 53)	1 (8, 0 to 35)	8 (67, 39 to 86)
2.2	Were predictor assessments made without knowledge of outcome data?	61 (65, 55 to 74)	2 (2, 1 to 7)	31 (33, 24.3 to 43)	7 (58, 32 to 81)	1 (8, 0 to 35)	4 (33, 14 to 61)
2.3	Are all predictors available at the time the model is intended to be used?	74 (79, 69 to 86)	2 (2, 1 to 7)	18 (19, 13 to 28)	8 (67, 39 to 86)	0 (0.0)	4 (33, 14 to 61)
Outcome							
3.1	Was the outcome determined appropriately?	73 (78, 68 to 85)	4 (4, 1 to 10)	17 (18, 11 to 27)	8 (67, 39 to 86)	0 (0.0)	4 (33, 13.8 to 61)
3.2	Was a prespecified or standard outcome definition used?	76 (81, 72 to 88)	3 (3, 1 to 9)	15 (16, 10 to 25)	10 (83, 55 to 95)	0 (0.0)	2 (17, 4.7 to 45)
3.3	Were predictors excluded from the outcome definition?	63 (67, 57 to 76)	6 (6, 3 to 13)	25 (27, 19 to 36)	8 (67, 39 to 86)	0 (0.0)	4 (33, 13.8 to 61)
3.4	Was the outcome defined and determined in a similar way for all participants?	78 (83, 74 to 89)	6 (6, 3 to 13)	10 (11, 6 to 19)	7 (58, 32 to 81)	1 (8, 1 to 35)	4 (33, 13.8 to 61)
3.5	Was the outcome determined without knowledge of predictor information?	43 (46, 36 to 56)	7 (7.4, 3.7 to 15)	44 (47, 37 to 57)	3 (25, 9 to 53)	1 (8, 1 to 35)	8 (67, 39.1 to 86)
3.6	Was the time interval between predictor assessment and outcome determination?	67 (71, 61 to 79)	2 (2, 1 to 7)	25 (27, 19 to 36)	7 (58, 32 to 81)	1 (8, 1 to 35)	4 (33, 14 to 61)
Analysis							
4.1	Were there a reasonable number of participants with the outcome?	36 (38, 29 to 48)	45 (48, 38 to 58)	13 (14, 8 to 22)	5 (42, 19 to 68)	4 (33, 14 to 61)	3 (25, 9 to 53)
4.2	Were continuous and categorical predictors handled appropriately?	22 (23, 16 to 33)	20 (21, 14 to 31)	52 (55, 45 to 65)	0 (0.0)	1 (8, 1 to 35)	11 (92, 65 to 100)
4.3	Were all enrolled participants included in the analysis?	48 (51, 41 to 61)	21 (22, 15 to 32)	25 (27, 19 to 36)	6 (50, 25 to 75)	2 (17, 5 to 45)	4 (33, 14 to 61)
4.4	Were participants with missing data handled appropriately?	14 (15, 9 to 24)	47 (50, 40 to 60)	33 (35, 26 to 45)	2 (17, 5 to 45)	6 (50, 25 to 75)	4 (33, 14 to 61)
4.5	Was selection of predictors based on univariable analysis avoided?	63 (67, 57 to 76)	18 (19, 13 to 28)	13 (14, 8 to 22)		NA	
4.6	Were complexities in the data (e.g., censoring, competing risks, sampling of control participants) accounted for appropriately?	42 (45, 35 to 55)	19 (20, 13 to 29)	33 (35, 26 to 45)	5 (42, 19 to 68)	2 (17, 5 to 45)	5 (42, 19 to 68)
4.7	Were relevant model performance measures evaluated appropriately?	14 (15, 9 to 24)	23 (25, 17 to 34)	57 (61, 51 to 70)	3 (25, 9 to 53)	2 (17, 5 to 45)	7 (58, 32 to 81)
4.8	Were model overfitting and optimism in model performance accounted for?	50 (53, 43 to 63)	32 (34, 25 to 44)	12 (13, 8 to 21)		NA	

Table S2. PROBAST Signaling questions for model development and validation analyses in 94 included prognostic studies

		Developed models (n=94)			External validations (n=12)		
		Yes or probably yes *n* (%; 95% CI)	No or probably no *n* (%; 95% CI)	No information *n* (%; 95% CI)	Yes or probably yes *n* (%; 95% CI)	No or probably no *n* (%; 95% CI)	No information *n* (%; 95% CI)
Participants							
1.1	Were appropriate data sources used, e.g. cohort, RCT or nested case-control study data?	70 (75, 65 to 82)	21 (22, 15 to 32)	3 (3, 1 to 9)	10 (83, 55 to 95)	2 (17, 5 to 45)	0 (0.0)
1.2	Were all inclusions and exclusions of participants appropriate?	57 (61, 51 to 70)	12 (13, 8 to 21)	25 (27, 19 to 36)	5 (41, 19 to 68)	0 (0.0)	7 (58, 32 to 81)
Predictors							
2.1	Were predictors defined and assessed in a similar way for all participants?	63 (67, 57 to 76)	14 (15, 9 to 24)	17 (18, 12 to 27)	3 (25, 9 to 53)	1 (8, 1 to 35)	8 (67, 39 to 86)
2.2	Were predictor assessments made without knowledge of outcome data?	61 (65, 55 to 74)	2 (2, 1 to 7)	31 (33, 24 to 43)	7 (58, 32 to 81)	1 (8, 1 to 35)	4 (33, 14 to 61)
2.3	Are all predictors available at the time the model is intended to be used?	74 (79, 69 to 86)	2 (2, 1 to 7)	18 (19, 13 to 28)	8 (67, 39 to 86)	0 (0.0)	4 (33, 14 to 61)
Outcome							
3.1	Was the outcome determined appropriately?	73 (78, 68 to 85)	4 (4, 2 to 10)	17 (18, 12 to 27)	8 (67, 39.to 86)	0 (0.0)	4 (33, 14 to 61)
3.2	Was a prespecified or standard outcome definition used?	76 (81, 72 to 88)	3 (3, 2 to 9)	15 (16, 10 to 25)	10 (83, 55 to 95)	0 (0.0)	2 (17, 5 to 45)
3.3	Were predictors excluded from the outcome definition?	63 (67, 57 to 76)	6 (6, 3 to 13)	25 (27, 19 to 36)	8 (67, 39 to 86)	0 (0.0)	4 (33, 14 to 61)
3.4	Was the outcome defined and determined in a similar way for all participants?	78 (83, 74 to 89)	6 (6, 3 to 13)	10 (11, 6 to 19)	7 (58, 32 to 80.7)	1 (8, 1 to 35)	4 (33, 14 to 61)
3.5	Was the outcome determined without knowledge of predictor information?	43 (46, 36 to 56)	7 (7, 4 to 15)	44 (47, 37 to 57)	3 (25, 9 to 53.2)	1 (8, 1 to 35)	8 (67, 39 to 86)
3.6	Was the time interval between predictor assessment and outcome determination?	67 (71, 61 to 79)	2 (2, 1 to 7)	25 (27, 19 to 36)	7 (58, 32 to 80.7)	1 (8, 1 to 35)	4 (33, 14 to 61)
Analysis							
4.1	Were there a reasonable number of participants with the outcome?	36 (38, 29 to 48)	45 (48, 38 to 58)	13 (14, 8 to 22)	5 (42, 19 to 68.0)	4 (33, 14 to 61)	3 (25, 8.9 to 53)
4.2	Were continuous and categorical predictors handled appropriately?	22 (23, 16 to 33)	20 (21, 14 to 31)	52 (55, 45 to 65)	0 (0.0)	1 (8, 1 to 35)	11 (92, 65 to 100)
4.3	Were all enrolled participants included in the analysis?	48 (51, 41 to 61)	21 (22, 15 to 32)	25 (27, 19 to 36)	6 (50, 25 to 75)	2 (17, 5 to 45)	4 (33, 14 to 601)
4.4	Were participants with missing data handled appropriately?	14 (15, 9 to 24)	47 (50, 40 to 60)	33 (35, 26 to 45)	2 (17, 5 to 45)	6 (50, 25 to 75)	4 (33, 14 to 61)
4.5	Was selection of predictors based on univariable analysis avoided?	63 (67, 57 to 78)	18 (19, 13 to 28)	13 (14, 8 to 22)		NA	
4.6	Were complexities in the data (e.g., censoring, competing risks, sampling of control participants) accounted for appropriately?	42 (45, 35 to 55)	19 (20, 13 to 29)	33 (35, 26 to 45)	5 (42, 19 to 68)	2 (17, 5 to 45)	5 (42, 19 to 68)
4.7	Were relevant model performance measures evaluated appropriately?	14 (15, 9 to 24)	23 (25, 17 to 34)	57 (61, 51 to 70)	3 (25, 9 to 53)	2 (17, 5 to 45)	7 (58, 32 to 81)
4.8	Were model overfitting and optimism in model performance accounted for?	50 (53, 43 to 63)	32 (34, 25 to 44)	12 (13, 8 to 21)		NA	
